# Losing half the crown hardly affects the stem growth of a xeric southern beech population

**DOI:** 10.1038/s41598-025-90061-9

**Published:** 2025-02-17

**Authors:** Ernesto J. Reiter, Robert Weigel, Christoph Leuschner

**Affiliations:** 1 Plant Ecology and Ecosystems Research, Albrecht von Haller Institute for Plant Sciences, University of Göttingen, Göttingen, Germany; 2 Ecological-Botanical Garden, University of Bayreuth, Bayreuth, Germany

**Keywords:** Crown die-back, Dendrochronology, Drought, Growth trend, *Nothofagus Pumilio*, Patagonia, Plant sciences, Ecology

## Abstract

Globally, forest ecosystems face increasing climate warming-driven stress. Crown dieback is commonly used as an indicator of declining tree vitality and is closely related to reduced stem radial growth rates. In a xeric northern Patagonian *Nothofagus pumilio* population, in which the majority of trees possess damaged crowns, we explored the relationship between percent crown damage and growth trends (basal area increment, BAI), interannual growth variability, and the climate sensitivity of growth. The majority of trees show stable BAI since about 1940 despite 5 to > 50% crown damage, which ranges from dieback of small branches to the presence of decades-old snagged branches. A minority of trees with more severe crown damage (> 50 to 95%) show continued growth decline during the last 80 years, but have not yet died. Crown damage was the best predictor of the BAI trend which turned negative at about 50% damage. Stronger damaged trees showed a higher growth sensitivity to summer heat and drought. Thus, the health of this population is apparently not threatened by crown damage up to 50%. Rather, trees might profit from the reduced foliage area, allowing them to stabilize their water relations and maintain stable but fairly slow growth in a drying climate.

**Supplementary Information:**

The online version contains supplementary material available at 10.1038/s41598-025-90061-9.

## Introduction

Forest decline encompasses the premature, gradual or rapid deterioration of forest ecosystem vitality, where a significant proportion of individuals generate a collective reduction of forest health and its resilience to environmental changes^1^. Declining forest health includes reduced productivity^2^, decreased stress resilience^3^, the alteration of nutrient cycles^4^, changes in species composition^5^, and reduced carbon (C) storage and climate regulation^6^, which can generate positive feedback to climate warming^7^.

When a given abiotic or biotic agent alters the normal metabolic activity of a tree, it often leads to immediate growth reduction, and depending on stress intensity, duration and frequency, to subsequent recovery or continued vitality decline^8–10^. The etiology of declining trees often involves the interaction of several factors, rather than a single leading cause, that predispose and incite growth decline, and the action of contributing factors that eventually lead to tree death^11^. The question of how long declining trees can sustain reduced growth before they die, remains largely unanswered, since the process can take from a few months to more than a century and depends on the physiological constitution, age, stand density, and legacy effects of former stress events, and varies greatly among the species^9,12,13^. Studies addressing this knowledge gap are crucial for understanding the potential impacts of climate change on forest health, and whether they may eventually lead to shifts in species composition that alter ecosystem functioning.

Various studies have identified crown damage as a powerful predictor of tree vitality loss and subsequent mortality^14–16^. Early stages of crown damage often manifest in the form of leaf discoloration, pre-senescent defoliation, and dieback of small branches and twigs^17,18^. In more advanced stages of damage, thicker branches may lose their hydraulic capacity, causing the dry-down of crown parts and enhancing the susceptibility to mechanical injuries and biological attack^4,19,20^. Crown damage can reduce photosynthetic carbon gain^21,22^, which further accelerates tree vitality decline and often leads to a death spiral^15,20,23^.

Another weakness symptom is long-term growth decline of the stem, often in combination with increased inter-annual radial stem growth variability and elevated climate sensitivity of growth, which is frequently associated with crown defoliation^9,18,24^. Moreover, dendrochronological studies have consistently demonstrated that trees characterized by long-term growth decline are more vulnerable to further stresses such as subsequent droughts^18^ and biotic agents^25,26^. Growth decline, increased growth variability and elevated climate sensitivity of growth evidenced in tree rings also may lead to increased mortality, especially after recurrent drought events^9,27^. However, the relation between morphological weakness indicators, growth decline and mortality is not in all cases tight and often difficult to predict^12,28,29^.

Long-term acclimation of trees to a warmer and drier climate may include both changes in physiology and morphological adjustments in leaf area, root mass and the hydraulic system^30,31^. Leaf area reduction can save water and thus improve the tree’s water status, but it is not well known, how old trees achieve this reduction. One possible mechanism could be to sacrifice part of the crown during drought episodes, as has been observed in woody plants of dry forests^32^, but its significance is not well studied for temperate tree species.

In the temperate forest region of southern South America, partial crown die-back and standing dead trees are a characteristic feature of *Nothofagus* forests at the xeric margin of their distribution range^19,33,34^. Even though the etiology of *Nothofagus* decline is not well understood, increasing evidence suggests that severe drought events are widespread triggers of growth decline and crown damage^19,35,36^. In northern Patagonia, in the rain-shadow east of the Andes, the broadleaf deciduous *Nothofagus pumilio* (Poepp. & Endl.) Krasser, locally known as Lenga, is an important native tree species at montane to subalpine elevation, which is capable of tolerating frost and heavy snow loads in winter and withstanding summer droughts^37,38^. *N. pumilio* is a moderately shade-tolerant species^39^, which forms monospecific stands of tall-growing trees at montane elevation and krummholz scrub at the tree line under a wide range of precipitation levels from > 5000 mm yr^− 1^ in the moister west to < 500 mm yr^− 1^ in the drier east^34^. Natural *N. pumilio* forests typically are composed of small-scale mosaics of patches differing in tree age that developed after small-scale disturbances mainly due to senescence or wind-throw^34,40^.

The species occupies a broad climatic niche ranging from a Mediterranean-type climate with a drought period in summer at 35 °S to a humid cool-temperate climate at 56 °S in Tierra del Fuego^41^. In accordance, several studies have demonstrated that *N. pumilio* populations are characterized by high phenological plasticity^37,42^ and considerable genotypic variation^43,44^, which likely has led to the development of different ecotypes and provenances. Consequently, forest structure and productivity, and the climate sensitivity of growth typically vary markedly with latitude^45^, but also along local precipitation gradients^40,46^.

While subalpine *N. pumilio* stands usually have shown enhanced growth with climate warming since the second half of the 20th century, more recent growth trends have turned negative in various regions, suggesting a gradual shift in the nature of *N. pumilio* growth limitation from cold-limited to drought- and heat-limited^45–48^. This shift mirrors the pronounced warming and drying trend of the climate in northern Patagonia east of the Andes since the late 1970s^49^. Drought-induced growth decline and increased mortality of *N. pumilio* have been reported not only from dry^35,50^ but also from humid subalpine stands^47^. In declining *N. pumilio* stands, trees with severe crown damage often co-exist with healthy trees^19,51^, in a similar manner as was observed in declining northern hemispheric broadleaf forests (e.g. in European beech, *Fagus sylvatica* L^17,29^). A major driver of crown damage and death in *N. pumilio* stands in recent time is defoliation by the moth *Ormiscodes amphimone* (Lepidoptera, Saturniidae)^52–54^. Declining *N. pumilio* trees often show a certain degree of crown damage, signs of moth or, less frequently, bark beetle, and woodpecker attack, and infection by hemi-parasitic plants such as *Misodendrum* (Misodendraceae) species^34,35,55^.

Since crown damage is often different among the trees in declining *N. pumilio* stands^15,34^, considerable within-stand variation in climate sensitivity and growth trends is to be expected. Consequently, mean growth trends, as are frequently reported in dendroecological studies, likely are obscuring such divergent growth trajectories among the individuals^51^. Moreover, the physiological response to short- and long-term climatic variability likely will differ between severely affected and healthy trees^56^, which requires the selection of detrending methods aimed at capturing both high- and low- frequency climate signals^57^.

**Fig. 1:**
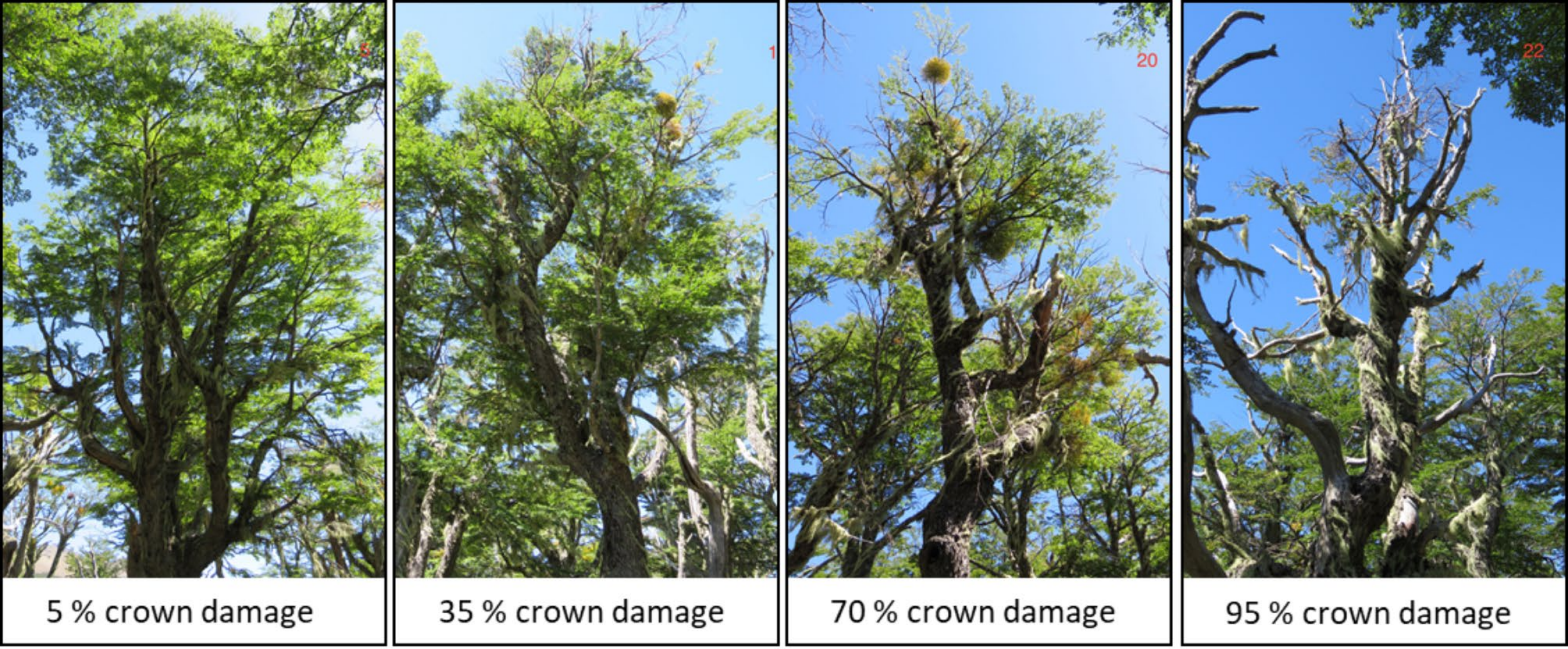
Visually assessed crown damage of four *N. pumilio* individuals in an old-growth forest at a xeric site in northern Patagonia, ranging from low (< 25%) to moderate (26 to 65%) and severe (66 to < 95%) crown damage.

In this case study, we explore the patterns of growth decline in an old-growth *N. pumilio* forest at a xeric site at upper montane to subalpine elevation in northern Patagonia and study their dependence on crown damage as well as tree size and age and stand density. We search for variation in growth patterns among the trees in the stand and assigned the trees to distinct groups with similar long-term basal area increment trends, in order to search for drivers that influence growth trend direction. Since the identification of climatic signals in tree-ring chronologies may be influenced by the applied detrending and standardization method, we compare the outcome of various detrending and standardizing methods in an attempt to discern different climate sensitivities of growth in tree individuals with contrasting growth trends. By linking growth performance to crown damage (see Fig. 1), we contribute to the effort to employ visually assessed crown vitality as an indicator of long-term growth trends and vitality decline in temperate tree species. Three main questions guided our study: (1) Do trees affected by partial crown damage experience a long-term, continuous decline in radial growth? (2) Does the growth sensitivity to climatic drivers differ between trees with varying proportions of crown damage? (3) Is there a critical degree of crown damage beyond which growth shifts irreversibly to a negative trend?

## Results

### Main chronology and dominant patterns of tree growth

The dendrochronological analysis yielded 46 dated cores from 23 *N. pumilio* trees that varied in crown damage from 5 to 95% and thus covered the full range of crown vitality variation from healthy (only insignificant leaf loss and no dead branches) to nearly completely damaged (only very few branches with leaves present). The EPS of the site chronology was well above the 0.85 threshold, and the Rbar value close to 0.6 indicates moderate agreement among the tree-ring series of the stand (Table 1).

According to the PCA analysis, two different long-term growth trajectories were identified in the main BAI chronology; the corresponding two first PCA axes explained more than 70% of the total BAI variability in the stand (PC1: standard deviation: 3.61, variance explained: 58%; PC2: standard deviation: 1.76, variance explained: 13%; Table S2). Chronologies derived for the trees assigned to the two groups (PC1 and PC2) showed acceptable quality statistics values, even though the lower number of sample trees reduced the EPS and Rbar values of these subgroups (Table 1).

**Table 1:** Descriptive statistics of the site chronologies and of the two chronologies related to PC1 or PC2 as derived from the PCA analysis. EPS: expressed population signal, Rbar: mean inter-series correlation.

	Site chronology	PC1	PC2
No. trees (series)	23 (46)	17 (24)	6 (12)
Start year	1721	1721	1731
Series period (*n* < 5)	1761–2021	1769–2021	1890–2021
Rbar	0.587	0.482	0.549
EPS	0.970	0.941	0.879

**Fig. 2:**
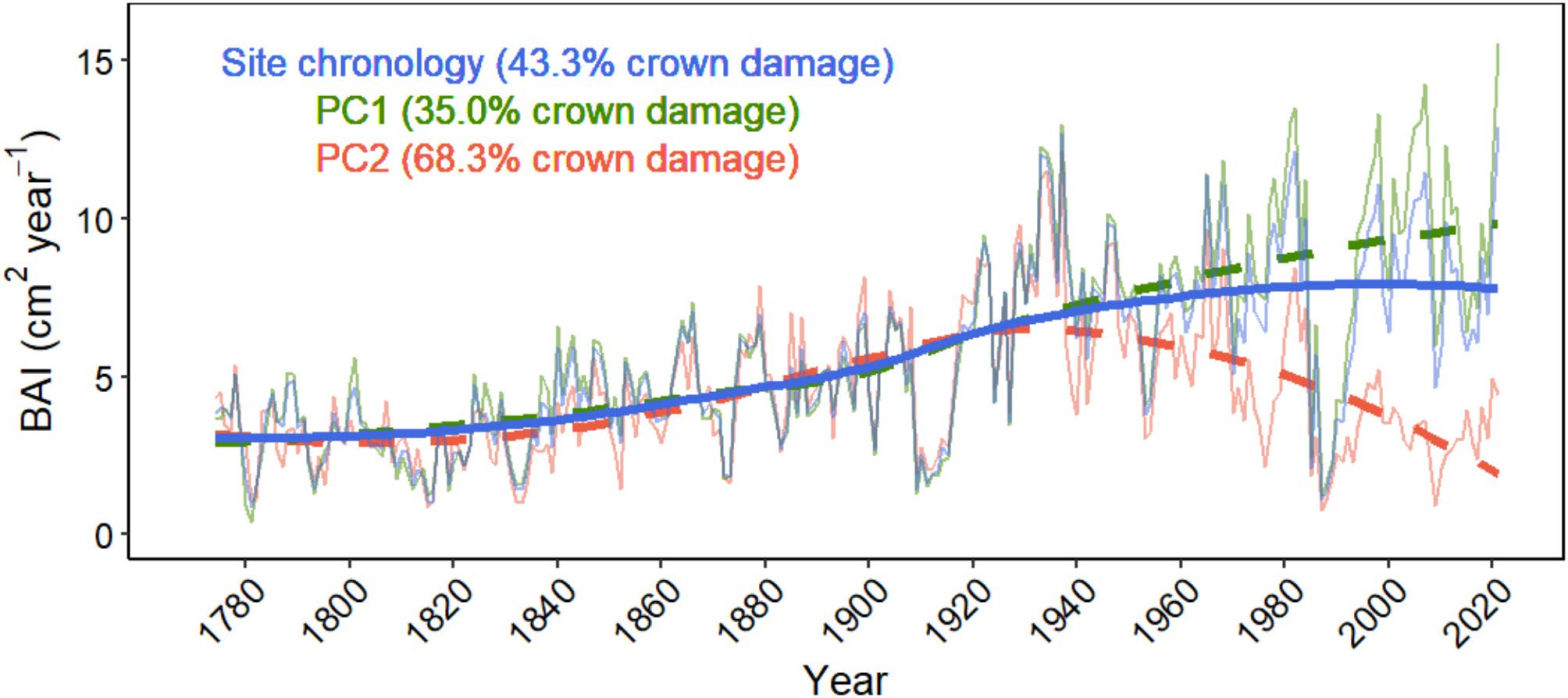
Basal area increment (BAI) of *N. pumilio* during the last 240 years according to the stand-level chronology (all 23 trees; blue solid line), and the chronologies of the trees associated to PC1 (green dashed line) and to PC2 (red dashed line). The mean estimated crown damage of the three groups is indicated in brackets.

**Fig. 3:**
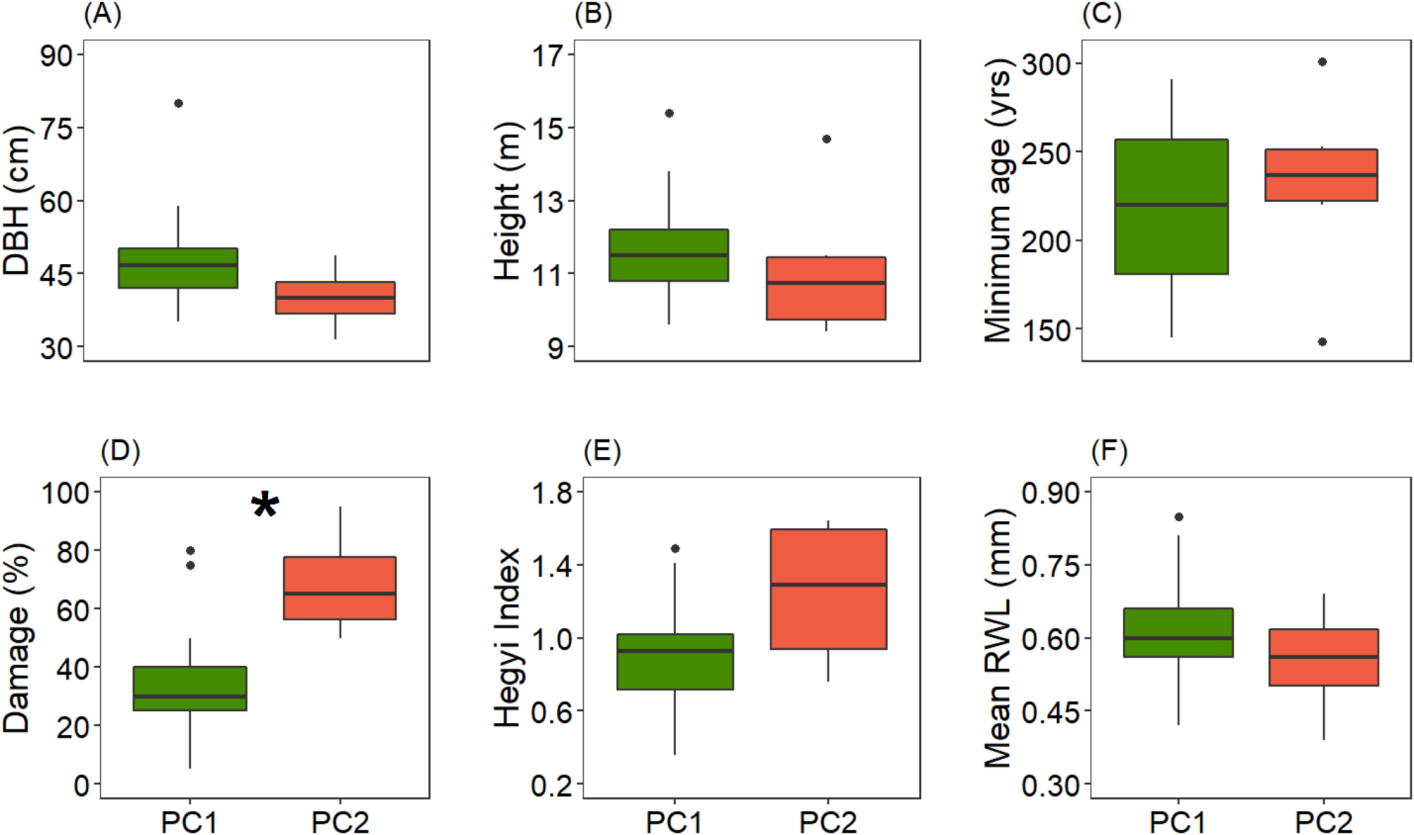
Six tree- and growth-related traits of the trees associated with the PC1 and PC2 groups derived from the principal component analysis of tree growth patterns. Shown are median, 25- and 75-percentiles (box), whiskers extending from the inter-quartile range (IQR) to the largest or lowest values up to 1.5 * IQR, and outliers (dots). DBH - diameter at breast height, Height – tree height, Damage – crown damage, Hegyi Index - Hegyi competition index, mean RWL - mean ring width length. The asterisk indicates a significant difference between means (*p* < 0.05). Number of trees: PC1 (17), PC2 (6).

All 23 trees have followed almost identical long-term BAI trajectories until about 1940. Since then, the growth trends of the trees in the two subgroups (PC1 and PC2) diverge, with the less-damaged trees of group PC1 (*n* = 17; Fig. 3D) roughly continuing their positive BAI trend (Tau = 0.21, *p* = 0.005) they had followed during the last 100 years (Fig. 2: green dashed line). In contrast, the trees of group PC2 with significantly more damaged crown (*n* = 6; Fig. 3D) turned in 1934 (according to the loess model; Fig. 2) to a negative BAI trend (Tau = -0.457, *p* < 0.001), which reduced growth rate from 1941 to 2021 to the half (Fig. 2: red dashed line). The site chronology (whole population) showed a positive BAI trend until 1940, which weakened afterwards to approach a stationary growth rate from 1980 onwards (Tau = 0.098, *p* = 0.191; Fig. 2: blue solid line).

The comparison of the two subgroups revealed no significant group differences in DBH, tree height, minimum age, Hegyi competition index, and mean ring width (Fig. 3).

## Possible drivers of different growth trends

**Fig. 4:**
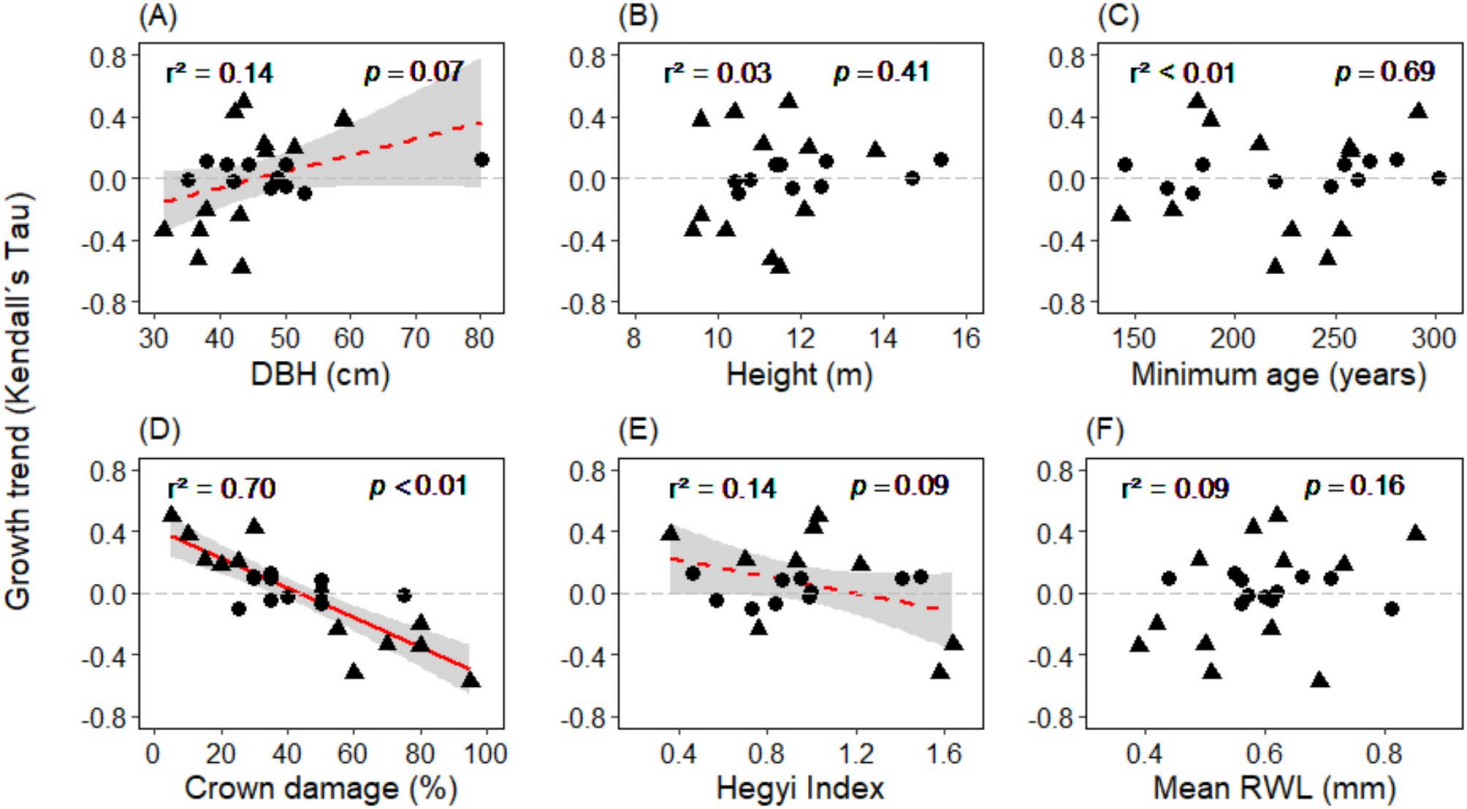
Dependence of the tree individuals’ long-term BAI trends in the 1941–2021 period (as expressed by Kendall’s Tau) on six tree- or growth-related traits. Red lines give significant (*p* < 0.05) least-squares regression with 95% confidence intervals. The dashed lines show marginally significant regression lines at *p* < 0.1. Triangles stand for trees showing significantly increasing or decreasing BAI trends (*p* < 0.05), dots for trees with no significant variation in growth trend.

There was only a marginally significant linear relationship of growth trend direction to DBH (smaller trees with more negative BAI trend) and competition intensity (more negative growth trends with higher competition intensity), and no relationship to tree height, minimum age, and mean ring width (RWL) (Fig. 4). In contrast, percent crown damage had a highly significant (*p* < 0.01) negative effect on the BAI trend direction (Fig. 4D). The growth trend turns negative (Tau < 0), when crown damage exceeds 43%. Significantly negative BAI trends (*p* < 0.05) where observed, when trees had ≥ 55% crown damage.

**Fig. 5:**
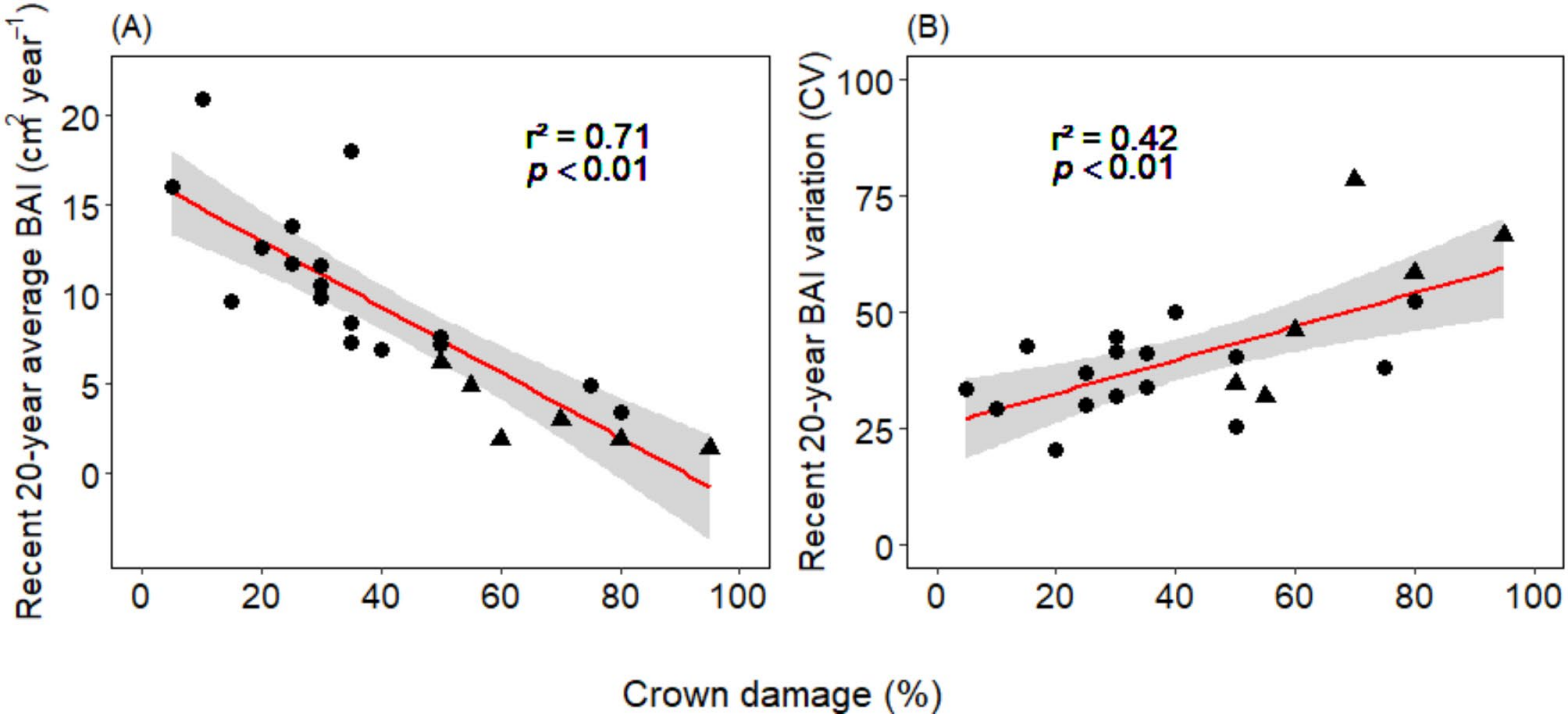
Dependence of (**A**) recent growth rate (BAI of the 2002–2021 period) and (**B**) inter-annual BAI variation in the 2002–2021 period as expressed by the coefficient of variation (CV) on crown damage in the 23 studied trees. Red lines give least squares regression lines with 95% confidence intervals. Dots stand for trees assigned to PC1, triangles for trees assigned to PC2 of the PCA.

The degree of crown damage largely explained the trees’ recent growth rates (mean BAI during the last 20 years) (Fig. 5A). Higher crown damage also seems to increase inter-annual variability in BAI as expressed by the coefficient of variation (CV) (Fig. 5B).

## Climate sensitivity of growth

**Fig. 6:**
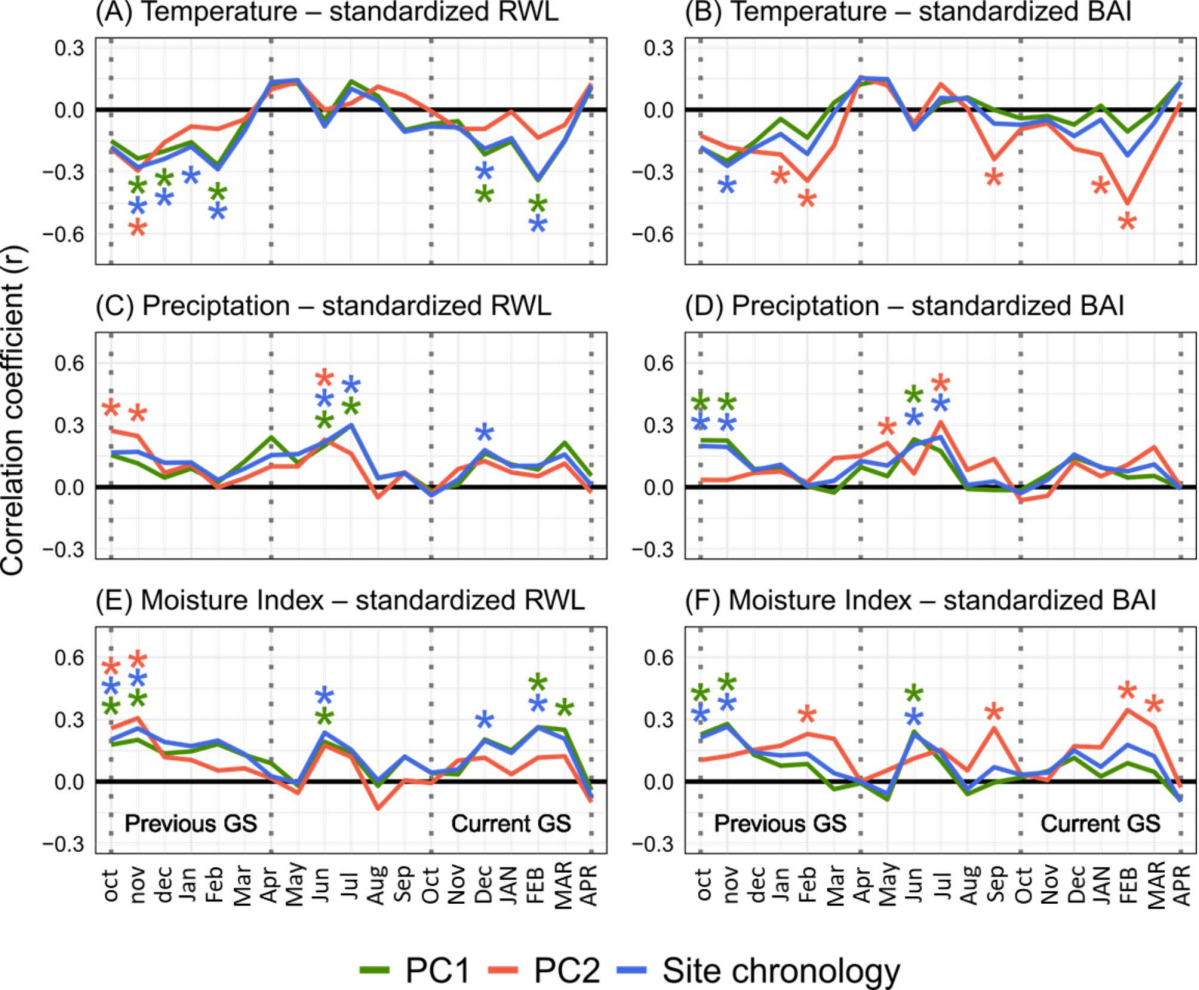
Pearson correlation coefficients of the relationship between standardized ring width length (RWL, left column) or basal area increment (BAI, right column) using the deviations of series mean, and departures of temperature, precipitation and moisture index of different months, for the stand-level site chronology (blue, all 23 trees) and the two subgroups of trees as derived in the PCA analysis (PC1 and PC2; green and red, respectively). Asterisks indicate significant correlations (*p* < 0.05). Months from oct to Apr represent the previous growing season, May to Sep the winter months, and Oct to APR the current growing season. GS – growing season.

Across all evaluated detrending methods, summer temperatures, winter and late spring precipitation, as well as previous growing-season moisture index largely drove the inter-annual variation in growth (Table S3). While these principal results remained fairly consistent across the different methods used for standardization and detrending the tree-ring series prior to calculating climate–growth relationships, the correlation coefficients and the number of significantly correlated months to annual growth rate were noticeably influenced by the method used (see Appendix II). Differences in coefficients were also observed when using either RWL or BAI as input data, with the more damaged trees (PC2 group) showing stronger climate signals in the BAI series (Fig. 6B, D and F), and the site chronology and the less damaged trees (PC1) showing stronger climate signals in the RWL series (Fig. 6A, C and E).

According to the correlation between climate variables and standardized RWL or BAI chronologies using the mean as a standardization method, growth was promoted by a moist climate and limited by elevated temperatures in early summer (December) (Fig. 6A and E). Later in summer, elevated temperatures further constrained growth, particularly during February, where the temperature sensitivity of growth was highest (Fig. 6A and B). Previous growing season’s climate influenced growth primarily through high temperature-limitation in late spring and summer, particularly in November (Fig. 6A and B). Growth was promoted by precipitation in previous year’s October and November (Fig. 6C and D), as it was by a higher moisture index especially in February (Fig. 6E and F). Winter precipitation during June and July, most probably as snow, had a positive effect on growth (Fig. 6C and D).

While the climate-growth correlation of the PC1 group plausibly followed largely that of the main chronology (as 17 of the 23 trees were identical), the climate sensitivity of the PC2 group trees differed in some aspects (Fig. 6). The negative effect on BAI of elevated temperature and the positive effect of a higher moisture index in recent mid-summer were more pronounced in the more damaged PC2 group, while the same effect on RWL was more prominent in the less damaged PC1 group.

**Fig. 7:**
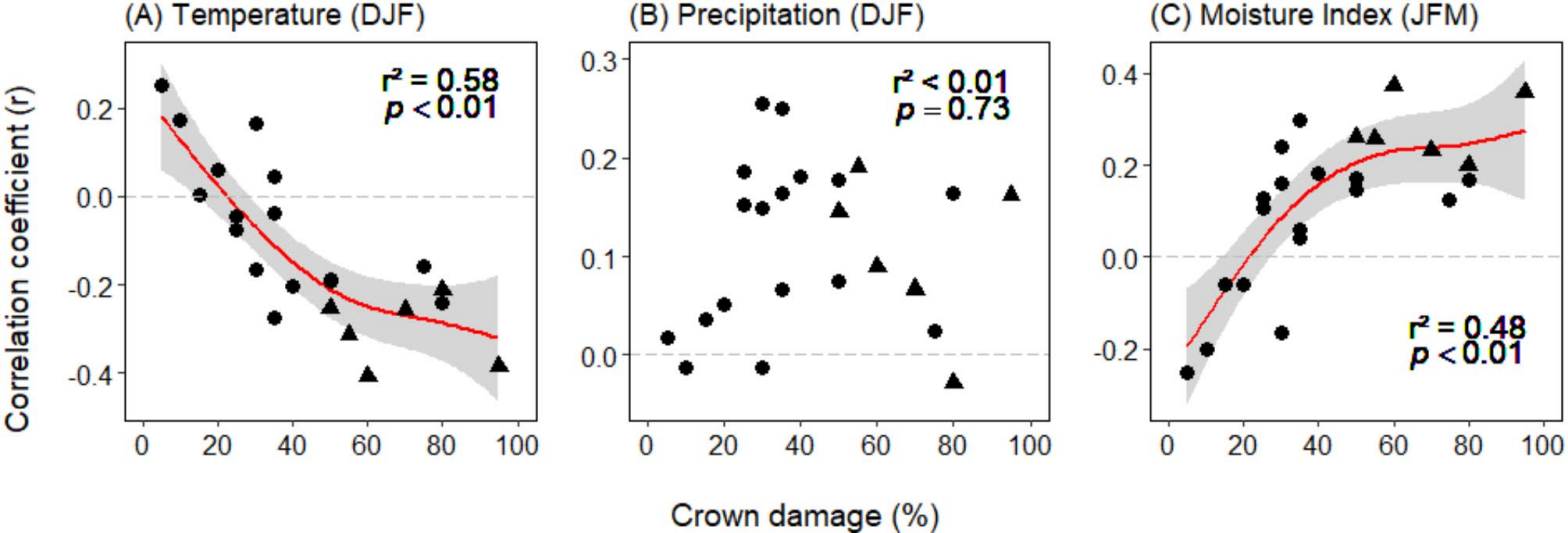
Average correlation coefficient (Pearson’s r) between the standardized BAI of the 23 trees and (**A**) temperature and (**B**) precipitation during December, January and February, and (C) Moisture Index (C) during January, February and March, in its dependence on crown damage of the trees. The red curves give the shape of significant relationships (*p* < 0.05) according to general additive models. Dots stand for trees assigned to PC1, triangles for trees assigned to PC2 of the PCA.

The correlation coefficients between the growth of individual trees and summer temperature (December – February; negative relationship) and summer moisture index (January – March; positive relationship) significantly increased with increasing crown damage (Fig. 7A and C). The related generalized additive model suggests that the percentage of crown damage is a significant predictor of the growth sensitivity to both climate factors, explaining 57.6% and 47.4% of the deviation from the mean, respectively. In contrast, the degree of crown damage had no significant influence on the growth sensitivity to summer precipitation (Fig. 7B).

From the explained variance, it appears that crown damage impacts growth primarily through the trees’ sensitivity to elevated early- to mid-summer temperatures (December – February) rather than through precipitation, as the r^2^ was highest for the temperature effect and close to zero for the precipitation effect (Fig. 7).

## Discussion

### Stable growth despite moderate crown damage

With more than 50% of trees having lost > 30% and up to 95% of foliage and branches, the visual assessment of crown vitality suggested for this *N. pumilio* stand near the species’ drought-limit a poor health status. To our surprise, this is not reflected in the overall basal area increment trends of the trees. Even though average crown damage was assessed to 43.3%, the mean growth trend of the population was positive in the 1941–1980 period and approached stable growth in the last 40 years. With a mean BAI of 7–8 cm^2^ yr^− 1^ in the last 80 years, the trees at this xeric site at upper montane to lower subalpine elevation grew not much slower than mature *N. pumilio* stands in more humid regions of northern Patagonia at this elevation (typically 8–12 cm^2^ yr^− 1^ in the 1940–2020 period^45,51^), where crown damage is less conspicuous. This points at a remarkable capacity of this tree population to maintain stable growth despite the loss of a large part of its leaf area in the past. In fact, the presence of snags and dead branches in the crown suggests that the majority of trees must have lost their leaf area in past decades, apparently without a sustained negative impact on growth rate. Some trees were even capable of increasing growth, despite considerable crown damage. This indicates that the studied population must be well adapted to the harsh climatic conditions at the study site, where cool and snow-rich winters often combine with episodic summer droughts^58^. In fact, the trees must be capable of directing a constant flow of carbohydrates to the stem cambium, even though annual carbon (C) gain most likely has decreased in the past due to a reduction in leaf area.

One possible explanation of this apparent decoupling of radial growth from the assumed photosynthetic C gain is that the trees might fix carbon in surplus of their requirement^59^, while growth likely is primarily limited by low temperatures in the cold season and episodic drought in summer^35,45,60^. If C were most of the time a non-limiting resource of tree growth^61,62^, then decreases in leaf area and C gain would not necessarily lead to growth reductions, but rather to less disposal of surplus C or to allocation shifts. In accordance, non-structural carbohydrate pools in *N. pumilio* trees were found to vary only moderately with age, tree height, growth rate and environmental conditions, suggesting that growth decline is more a consequence of shifts in C allocation patterns than of carbon depletion^63^. In the absence of data on changes in leaf area and photosynthetic activity in the past, such an explanation must remain speculative, however.

Another factor that might have contributed to the stable growth trends visible in our data is related to the position on the stems, where wood coring took place. We often found partially decayed and healthy sapwood in close contact within our increment cores, but these decayed areas in the core were consistently located closer to the rotten heartwood than to the younger, outermost rings near the bark. Since we cored stem sections without bark damage, and radial growth primarily occurs in the healthy parts of the stem wood, this procedure could bias basal area increment calculation, when being extrapolated to total stem cross-sectional area: If the area of rotten wood has increased over time, growth analyses in the healthy stem section could falsely indicate stable growth, even though total BAI of the stem has decreased. In this case, only analyses of cross-sections from the whole stem could provide accurate basal area increment estimates^64^. Further, dendrochronological study of *N. pumilio* trees suggested that stems with damaged bark might show over-proportional radial growth rates^15^, which could also lead to overestimation of basal area increment. However, since we avoided stems with damaged bark for coring, the latter phenomenon is probably less relevant in our study. Nevertheless, even if true growth rate has been overestimated in some of our trees, the long-term performance of the trees with up to 50% crown damage is astonishing, as the presence of old snags and missing branches suggests that damage must have occurred already decades ago. Moreover, the surviving trees have recovered from the severe decline episodes around 1910, 1975 and 1985. This implies a high capacity of the surviving *N. pumilio* trees to restore the functioning of crown, hydraulic system and root system after climatic, mechanic or biotic damage.

### Differential climate sensitivity of growth

Our climate-sensitivity analysis suggests that the growth of trees with decreasing growth trends (> 50% crown damage; PC2 group) seems to be more sensitive to elevated summer temperatures and lower moisture in summer, than that of the less damaged trees of the PC1 group. This is in agreement with the general increase in correlation strength observed between BAI and temperature (negative relation) and moisture index (positive relation) with increasing crown damage (Fig. 7). It also fits earlier study results that reported severe drought events after periods of favorable growth to be the main predisposing and inciting factor of *N. pumilio* stand-level decline^23,35,65^.

Our comparison of five standardizing and detrending methods (plus the raw data approach) and the use of either RWL or BAI data reveals that different methods lead to somewhat different pictures of climate-growth relationships. While the relative importance of various climatic drivers of growth was not different in these different runs, analyses based on BAI indicated a greater temperature and moisture sensitivity of the more damaged PC2 trees, while RWL-based analyses suggested greater sensitivity of the PC1 trees. This discrepancy may relate to the fact that the climate signal in the growth patterns likely is more clearly visible in BAI chronologies, which are less influenced by geometrical age and size effects with increasing stem diameters than are ring-width chronologies^66^. In correspondence, BAI chronologies were found to reflect climate-driven growth trends of *N. pumilio* better than RWL chronologies^35^.

In northern Patagonian *N. pumilio* stands, more than half of the inter-annual growth variability that was related to temperature fluctuation was found to be associated with periodicities longer than 6 years, i.e. reflecting low-frequency climate signals^60^. Detrending of tree-ring and climate series tends to enhance high-frequency climate signals, while it reduces the influence of long-term growth trends and low-frequency signals, thus strengthening annual climate–growth relationships^67,68^. However, when low-frequency climate signals are exerting an important influence on annual growth variability, as is often the case in woody plants in subalpine environments^57,60^, detrending of ring-series and climate data might partly erase the cumulative effect of climate on tree growth, which can unintentionally blur or wipe out signals of climate change on growth^69^. Different detrending methods will thus conserve high- or low-frequency climate signals in growth chronologies to a different extent, which is another explanation of the non-identical outcomes of the different detrending methods explored here.

## Possible causes of long-term growth decline

In contrast to the majority of trees in the stand, trees with > 50% crown damage showed consistent BAI declines during the last 80 years, leading to a halving of growth rate until the year 2021. Since damage is significantly higher than in the trees with stable BAI, crown damage in the more distant past and subsequent impairment of metabolic activity is the most likely driver of reduced growth.

The long-term BAI chronologies demonstrate that negative growth departures occurred between 1935 and 1985 synchronously in the more damaged PC2 and the less damaged PC1 group of trees, suggesting that climatic or stand-level biotic stressors were active rather than asymmetric competition, age or drivers related to local edaphic stress (e.g. shallow, drought-affected soil patches). However, growth recovery was apparently different between the PC1 and PC2 trees, with the former (but not the latter) being able to restore growth completely after stress episodes. The early 20th century was a period of favorable growth conditions (generally warmer temperatures and increasing atmospheric CO_2_ concentration with no marked drought year; Fig. S1;^70^), when the then already more than 150-year-old trees reached the highest growth rates in their lifespan, which presumably were associated with further canopy expansion. Later in the decade, the moderate El Niño event in 1937-1939^71^ with exceptionally low mean annual temperatures and elevated precipitation in late spring, which is characteristic for El Niño years in the region^72^, must have decreased growth, and plenty of late-spring snow might have harmed the trees through snow-breakage or late frost events^60^. In the subalpine belt, the growth of *N. pumilio* is known to be limited by cold and wet conditions during early spring^45–47^, although the interaction between temperature and other growth-influencing factors such as late frosts, snow cover duration and growing season length may complicate the determination of key growth-limiting factors^60,73^.

While it is impossible to reconstruct the precise causes that have pushed the PC2 trees to a continued negative BAI trend in the past, it is likely that any damage to crown, hydraulic system or root system in that time was repaired to a lesser degree in the PC2 than in the PC1 trees. Subsequent stress events were encountered in 1954/1954 (a pronounced La Niña event with warmer and drier springs^72^) and 1970, which hampered the recovery of the PC2 trees even further and enhanced the negative BAI trend. Because the 1930s to 1970s were (with a few exceptions, i.e., 1943-44) cooler and moister than the decades after 1980, drought and heat are a less likely driver of growth decline in this period, suggesting the involvement of additional factors. Attack by pest organisms is a possible agent, which became visible especially in 1985 in a regional outbreak of the moth *Ormiscodes amphimone* (Lepidoptera: Saturniidae), whose caterpillars defoliated thousands of hectares of *N. pumilio* forest in northern Patagonia^34,54^. Even though *N. pumilio* is able to survive complete defoliation by *O. amphimone*, aided by the formation of more acquisitive and also more herbivore-resistant secondary leaves^53^, subsequent recovery was obviously incomplete in the already weakened PC2 trees, but not in the PC1 trees. This severe defoliation event was related to the warmer and drier episode that started with the 1976 Pacific Decadal Oscillation shift, when a decadal period of El Niño-dominated years changed to La Niña-domination^74^. Simultaneously, the Southern Annular Mode (SAM) has largely remained in a positive phase since then^75^ and the Southern Pacific Subtropical Anticyclone (SEPA) and with it the cold Humboldt current system weakened, dampening the prevailing westerly winds that bring precipitation to Patagonia south of 40°S^49^, causing more frequent summer droughts also at subalpine elevation. Even in the moister Chilean Andes, a general reversal of *N. pumilio* growth to a negative trend after the 1970s has been observed in tree-line populations^76^. The negative growth trend since about 1980 prevails despite an increase in intrinsic water use efficiency, and is related to a decrease in stomatal conductance as evidenced by increasing foliar δ^18^O signatures^48^. In our stand, the relatively warm and dry climate that is prevailing since the 1980s, together with the severe defoliation event in 1985 and presumed crown damage as a legacy of former stress events, has obviously pushed the PC2 trees toward further growth decline.

Some evidence suggests that the sudden dieback of some branches or parts of the crown in xeric populations of *N. pumilio* is caused by hydraulic failure^44,77^, as was observed during extreme hot droughts in other temperate broadleaf trees such as European beech as well^78^. In the absence of physiological measurements, however, it must remain open, whether partial hydraulic failure, root system damage, a reduced photosynthetic capacity of the crown, defoliation by insect attack or other mechanisms have driven the growth decline.

## Crown damage as a vitality indicator in N. Pumilio

A considerable body of evidence demonstrates for temperate trees a rather tight relation between the degree of drought-induced crown defoliation or the proportion of dead branches in the crown and continued negative growth trends^14,18,22^. Moreover, both visual crown damage and negative growth trends are increasingly used as early-warning signs of eventual mortality^9,17,19^. In agreement, crown damage has been identified as a fairly reliable indicator of a negative growth trend in *N. pumilio*^15^. However, in that study, 25% of the sampled trees showed growth decline without signs of crown damage. Our results principally support the value of crown damage as an indicator for long-term negative growth trends. In our stand, crown damage was with 70% of explained variance more closely associated with a negative BAI trend than any other stand structural or growth-related variable. On the other hand, 10% of the trees in the PC1 group showed stable recent BAI trends, despite having lost more than 50% of their crown. Thus, the indicative value of crown damage to predict growth trends may in part be obscured by the fairly high regeneration potential after crown damage of *N. pumilio*.

Negative growth trends have been successfully used as early-warning sign of later tree death in northern Patagonian *N. pumilio* forests^50^. In accordance, the occurrence of rotten stems has been recorded in declining *N. pumilio* stands^79^. The probability of death was consistently explained not only by low growth rates six years prior to death, but also by negative BAI trends in the last 30–45 years before death^50^. Since only few dead trees are present in our stand despite poor canopy health, we cannot verify the generalizability of this pattern. The number of lying or standing dead trees observed in our stand does not exceed the numbers expected for a more than 250-year-old natural *N. pumilio* forest due to biological senescence. Despite poor crown conditions, a stand decay has not yet started.

However, we cored only the surviving trees and did not sample the trees that have died in the last decades. Consequently, our mean chronologies give a too optimistic picture of the growth performance of the entire population and its sensitivity to the climatic changes that have happened in the last five decades. It is likely that some weaker individuals have died in the past decades, and the surviving trees represent the more vital and probably better adapted individuals of the population. Thus, we assume that the stress imposed by climate change has selected for the fitter individuals in this population, thereby increasing the probability of survival of the population in a more stressful climate.

For the present tree individuals, we can conclude that the observed 80-yr-long negative BAI trend has not yet led to death in these trees, suggesting that certain *N. pumilio* trees in high-elevation stands can survive in stressful environments far longer than was previously assumed. It appears that the lethal drought or heat stress threshold has not yet been reached by the PC2 trees.

### Drought acclimatization through leaf area reduction?

Our study in a drought-affected high-elevation *N. pumilio* stand demonstrates that severe crown die-off does in temperate tree species not necessarily cause death within a time horizon of several decades. The relatively slow-growing, stress-tolerant *N. pumilio* resembles in certain aspects the long-lived, highly drought-resistant conifers of semi-arid regions (such as *Pinus aristata* in the South-western U.S.), which can survive severe drought damage even for millenia^80^. The capacity of *N. pumilio* to sustain or even increase growth despite considerable crown damage (as demonstrated by the PC1 trees) may indicate that the species is capable of long-term morphological adjustment to increasingly dry (and hot) summer conditions.

Long-term stable growth under a markedly reduced leaf area suggests that the damaged trees may have profited from the leaf area and transpiration reduction by being able to adjust their water demand to the long-term deterioration of water availability in summer. Such a shift to a more conservative resource use strategy could have stabilized tissue water status in the remaining foliage and stem cambium and roots in a drying climate, which in turn enabled to maintain growth rate at a low but ‘safe’ rate. If so, crown size reduction due to drought damage would then represent a damage-driven acclimatization to less favorable growth conditions, rather than a potentially lethal threat to vitality. Such a morphological response would also correct any structural overshoot of crown size and leaf area that may have happened during more favorable growth periods in the past, as was probably the case during peak growth in the early 1930s. Further studies have to deepen our understanding of plastic crown morphological and leaf area adjustments to changing climatic conditions. In particular, it has to be proven that crown size reduction can indeed be acclimative, i.e. benefits the tree in the longer run. Further, future research has to show which tree species, and under what conditions, are capable of reducing leaf area to sustain growth in a more stressful climate.

## Materials and methods

### Study site and sampling procedure

The study site is located in northern Patagonia at an elevation of 1404 m a.s.l. on the Argentinian eastern slopes of the Andes south of Bariloche and north-east of El Bolsón (41.6803°S and 71.2283°W), close to the eastern margin of the Andean forest belt, where the forest passes over into the Patagonian steppe^81^. The climate is cool-temperate (mean annual temperature 5.2 °C; mean annual precipitation 754 mm according to the WorldClim2 data base^82^) with most precipitation that is transported with westerly air masses from the Pacific falling during winter. Summers are cool (mean temperature November – February 10.8 °C) and relatively dry (mean precipitation sum in November – February 75 mm), exposing trees to water deficits during the warmest months, which may limit productivity^40,81^. From the Chilean border in the west to the forest-steppe ecotone in the east, precipitation steeply decreases from > 3000 mm yr^− 1^ to 600 mm yr^− 1^ over less than 100 km distance^83^. The inter-annual climate variability of the region is largely influenced by the macroclimatic patterns shaped by the Antarctic Oscillation (AAO) and the El Niño-Southern Oscillation (ENSO)^74,75^. The natural vegetation at this elevation is forest dominated by *N. pumilio*, which progressively retreats in eastern direction to the cooler and more humid south- and east-facing slopes. More upslope at around 1500 m a.s.l., *N. pumilio* trees are replaced by dense lenga krummholz that forms the tree-line (ca. 1600 m a.s.l.). Towards lower elevations at ca. 1200 m a.s.l., *N. pumilio* forests abruptly pass over to post-fire shrublands dominated by the resprouting deciduous tree *Nothofagus antarctica* (Forst.) Oerst. At the study site, *N. pumilio* occurs on a slightly inclined slope (16°) in WNW exposition, which is in the region one of the most eastward (inland) stands of this species with northward (sun-exposed) exposition. The stand is a monospecific old-growth *N. pumilio* stand of ca. 12 m canopy height with no signs of forest management (as indicated by the absence of stumps^84^), a stem density of 416 ha^− 1^, and a total stand basal area of 53.7 m^2^ ha^− 1^. Canopy closure is fairly low with ca. 0.7 due to tree fall gaps and widespread crown damage. Few lying dead trees were present at frequencies characteristic for *N. pumilio* old-growth forests in the area^85^. Regeneration occurs patchily in dense cohorts that established in canopy gaps and have a height of 0.5–2 m. Most of the soil surface is covered by a herb layer composed of forbs, grasses and dwarf shrubs of low height, such as *Chiliotrichum diffusum* (G. Forst.) Kuntze, *Berberis microphylla* G. Forst., *Empetrum rubrum* Vahl ex Willd. and *Gaultheria mucronata* (L. f.) Hook. & Arn., as is characteristic for dry *N. pumilio* forests in Patagonia^86–88^. Tree regeneration and herb layer show signs of intensive browsing by wild cattle and exotic deer species.

A plot of 50 m x 50 m was demarcated in a patch of the stand where soil depth generally exceeded ca. 0.7 m in order to avoid growth impairment due to soil resource limitation in shallow profiles. Soil depth was systematically checked in the forest with a Pürckhauer soil auger, and a 1 m x 1 m soil pit was dug in the center of the plot to study soil conditions. Average soil depth in the plot was 81 cm, and the sandy-loamy volcanic soil material had a gravel content of 28.5% (particles > 2 mm). Forest structure, as described above, was inventoried in circular concentric plots in the center of the 50 m x 50 m plot. Diameter at breast height (ca. 1.3 m; DBH) and the height of all trees with DBH ≥ 7 cm were measured with a dendrometer band and a Vertex IV (Haglöf) height meter in a 400 m^2^ subplot, and the height and DBH of all trees with DBH ≥ 60 cm in a 1000 m^2^ subplot. No dead standing trees were present in this subplot.

### Target trees, crown damage estimation and wood coring

We selected the 25 dominant or co-dominant trees closest to the plot center, which lacked signs of rotten or damaged main stems. For each target tree, we registered DBH, total height and calculated the Hegyi competition index^89^ (CI) as an estimator of competition intensity in the direct vicinity of the tree, using the DBH of the target tree (dbh_i_), and the distance and DBH of its five nearest neighboring trees (dbh_j_ and dist_j_), according to Eq. (1):

1 $$\:{CI}_{i}=\sum\:_{j=1}^{n}\frac{{dbh}_{j}/{dbh}_{i}}{{dist}_{ij}}\:$$

All mature trees in the stand showed variable degrees of crown damage, ranging from the recent dieback of small branches with fine terminal twigs to larger dead branches and snags (Fig. 1). In order to estimate the percentage of crown damage in a reproducible way, we adapted the crown density health indicator of the US Forest Service’s Forest Inventory and Analysis scheme^90^ as follows: the proportion of crown damage is defined as the amount of branches and twigs as well as of foliage, buds and reproductive organs that are dead or broken as a fraction of the total visually reconstructed crown silhouette. The proportion of crown damage (in % of the total vertical crown cross-sectional area visible from the ground) was visually estimated by the three authors in the field and assigned to 5-percent classes. When discrepancies emerged between the three individual estimates, an average value was computed. Damage < 25% is defined as low, of 26–65% as moderate, and of 66 – >95% as severe.

From each target tree, two to three wood increment cores were extracted at breast height (ca. 1.3 m) in perpendicular directions using a 5 mm increment borer (Haglöf), while avoiding reaction wood. Coring took place during February 2022; so the last complete tree ring recorded was that of the growing season 2020–2021. The cores were fixed onto wooden mounts, sanded with increasingly finer grades of sandpaper (ranging from 200 to 1200 grains per cm^2^), and scanned at a resolution of 2400 dots per inch (dpi) using an Expression 11000XL scanner (EPSON).

### Dendrochronological analyses and assessment of growth patterns

The tree rings of the wood samples were visually dated using a Stemi 2000 stereomicroscope (Zeiss), and the ring widths were measured in the scanned images of the samples using the software CooRecorder (version 9.5, Cybis Elektronik & Data AB, http://www.cybis.se/forfun/dendro/). Cross-dating was done in accordance with the local master chronology “Diego de Leon” (Lavergne et al. 2015, available at the International Tree-Ring Data Bank, NOAA) by employing the software CDendro (version 9.5, Cybis Elektronik & Data AB, http://www.cybis.se/forfun/dendro/). The cross-dating of tree-ring series (ring width length; RWL) was validated through the correlation coefficient (*r* > 0.32), and Student´s t-value (T-test, t > 3.5)^91^ among series normalized by means of the P2YrsL method^92^, resulting in 46 acceptably dated cores. For the two or three dated series of a tree, one mean series was computed, resulting in 23 tree-ring series. For assessing long-term growth trends, basal area increment (BAI) was computed backwards step by step from diameter at breast height from the bark to the pith according to Eq. (2):

2 $$\:{BAI}_{t}=\:\pi\:\:\left({r}_{t}^{2}-{r}_{t-1}^{2}\right)$$

where *r* is the tree radius and *t* the year of ring formation. Analyzing growth trends through BAI reduces the geometrical influence of age and diameter on ring width^66^. As an average of the 23 individual tree-ring series, one chronology of BAI (unit: mm² yr^− 1^) and one dimensionless RWI chronology (ring width index) were computed with Tukey’s bi-weight robust mean. To assess the quality and reliability of the shared environmental signal across the sample, RWI series were computed from individual RWL series. This was done by detrending and standardizing the RWL series using a smoothing spline with a 50% frequency cut-off at 32 years^93^. This process removes age/size-related trends and the effects of individual disturbances, enhancing the inter-annual comparability of the tree-ring series^94^. RWI chronology quality was assessed through the Expressed Population Signal (EPS > 0.85)^8^ and mean inter-series correlation (Rbar value). All above-mentioned calculations were computed using the R package dplR^95^.

Dominant growth patterns in the population of 23 trees were identified using Principal Component Analysis (PCA) on the BAI data matrix, where years represent the environmental gradient (rows) and individual trees represent the variables (columns). The PCA transforms the annual BAI series of the trees into a smaller set of uncorrelated independent variables (components), with fewer axes capturing most of the common variance among all trees in the plot. The PCA was applied to the common overlapping period of all dated series (1879 to 2014). We retrieved individual series´ loadings for the first two principal components as representatives of two separate growth chronologies within the stand (PC1 and PC2; > 70% of cumulative variability explained). Each individual series was then assigned to that axis of the two, with which it correlated most. Subsequently, we tested whether stand- and growth-related traits (tree DBH and height, minimum age, crown damage, Hegyi competition index and mean RWL) differed significantly between the groups of trees either assigned to the PC1-chronology or the PC2-chronology. We used the Welch Two Sample t-test in the case of DBH, and the Mann–Whitney rank sum test for the other variables, according to data distribution and variance properties resulting from the unequal sample size of the PC1 and PC2 groups.

For these two tree groups identified in the PCA, RWI and BAI chronologies were computed as outlined above for the ring width series of the individual trees. Long-term BAI trends were tested for significance with the non-parametric Mann-Kendall trend test for the period 1941–2021. The strength and direction of growth trends was assessed by means of the Tau statistic in the Mann-Kendall test that assesses the correlation between data ranks and their chronological order^96^. Univariate regression models were built to analyze the relationship between long-term BAI trends (Kendall´s Tau) and tree characteristics (DBH, height, minimum age, crown damage, Hegyi competition index, and mean RWL). Since rotten stems were present in many old *N. pumilio* individuals, we took the number of rings present in the cores at 1.3 m height as the minimum age.

### Climate sensitivity of growth and its relationship with crown damage

To determine the climate influence on inter-annual growth variability, tree-ring chronologies were correlated with monthly temperature and precipitation data derived from six nearby weather stations (Table S1). The climatic records were standardized to the 30-year common reference period 1992–2021, and deviations in each series from their own mean were averaged to calculate regional means of temperature (3 stations) and precipitation departure (6 stations). For each station, the monthly data was standardized by calculating z-scores, where the average of the reference period is subtracted from each monthly value and is then divided by the standard deviation of the reference period. This produced monthly deviations from a mean of zero, expressed in terms of positive or negative standard deviations. While averaging temperature and precipitation departures (standard deviations), the contribution of each climate series to the regional mean is equally weighted^60^. For evaluating the impact of both climate variables simultaneously, a moisture index was calculated by subtracting the temperature from the precipitation departure^97^. In this approach, negative temperature departures increase positive precipitation departures, whereas positive temperature departures reduce negative precipitation departures. As a result, strongly positive anomalies indicate cold and wet years, while strongly negative anomalies indicate hot and dry years^98^.

The growth of tree species in subalpine, cold-limited environments does usually show a low sensitivity to annual climate fluctuations, whereas longer-term climatic trends spanning multiple years are better recognizable in the ring series^57,69^. Such a pattern has also been found in humid *N. pumilio* stands close to the tree-line^60^. Thus, for correlating annual growth with monthly standardized climate data, we first compared and evaluated different standardizing methods applied to the tree ring series, aiming to identify the method that maximizes both high- and low-frequency climate signals, while still minimizing the noise resulting from ageing and disturbances. We tested the standardizing methods by using both the RWL and BAI individual series as data input for building the chronologies. Details on the different tested detrending and standardization methods are given together with the results of this analysis in Appendix II. For further analysis, we proceed with the RWL and BAI series standardized by a fitted horizontal line that equals the mean of the series. This approach allows a straightforward standardization of the ring series of the studied slow-growing trees, which maximizes the recognition of long-term climate trend effects on growth, while still capturing most of the interannual variability. Further, the analyses based on BAI indicated an overall greater sensitivity than the RWL-based analyses. The climate-growth correlation coefficients were computed for the 1931–2021 period, the common period between the chronologies and the climatic records. The statistical significance of correlations was evaluated using a 1000-fold bootstrapped response function within the R package treeclim v2.0.6.0^99^. Since the regional growing season lasts approximately from October to March, spanning two calendar years in the southern hemisphere, we analyzed monthly climate-growth correlations for all months from previous growing season’s October to current growing season’s April (19 months over 3 calendar years).

For evaluating the influence of crown damage on the growth sensitivity to temperature, precipitation and the moisture index, we fitted generalized additive models (GAMs) between the climate-growth correlation coefficients (the relation between the individual tree’s standardized BAI and monthly climate variables) and crown damage in percent. The GAMs allow capturing non-linear relationships more flexibly than linear models, such as the detection of spatial trends via smooth functions of spatial covariates^100^.

## Electronic supplementary material

Below is the link to the electronic supplementary material.

Supplementary Material 1

Supplementary Material 2

## Data Availability

The replication data (ring width series, sampled trees data and regional climate) for the main analysis of this study can be found at the Göttingen Research Online repository at https://doi.org/10.25625/IQ2LB6.
