# Supplementary material for: Losing half the crown hardly affects the stem growth of a xeric southern beech population
Source: Sci Rep. 2025 Feb 17;15:5721. doi: 10.1038/s41598-025-90061-9 (PMC11832943; doi:10.1038/s41598-025-90061-9)
Supplement: Supplementary file 1 — Supplementary Material 1 [file 41598_2025_90061_MOESM1_ESM.pdf]

Appendix I

SUPPORTING INFORMATION

Tables

Table S1: Instrumental records of climatic data used for calculating the regional climate series.

Station	Latitude	Longitude	Variables	Period	Data source
Bariloche airport	-41.15111	-71.15750	Tmed; Pp	1931-2021	SMN (*)
Manso inferior	-41.55541	-71.78115	Pp	1963-2021	SNIH
Manso confluencia	-41.58690	-71.68363	Pp	1965-2021	SNIH
El Bolson airport	-41.94333	-71.53222	Tmed; Pp	1992-2021	SMN
El Maiten	-42.05084	-71.17180	Pp	1954-2021	SNIH
Esquel airport	-42.90590	-71.14550	Tmed; Pp	1931-2021	SMN (*)

(*) Data homogenized by Ricardo Villalba. Abbreviations: Tmax, maximum temperature; Tmin, minimum temperature; Tmed, mean temperature; Pp, precipitation; SMN, Servicio Meteorologico Nacional; SNIH, Servicio Nacional de Información Hidrica.

Table S2. Results of the Principal Component Analysis showing the first two components (70.3% of variance explained)

	PC1	PC2
Standard deviation	3.6161	1.7585
Proportion of Variance	0.5685	0.1344
Cumulative Proportion	0.5685	0.703

Figures

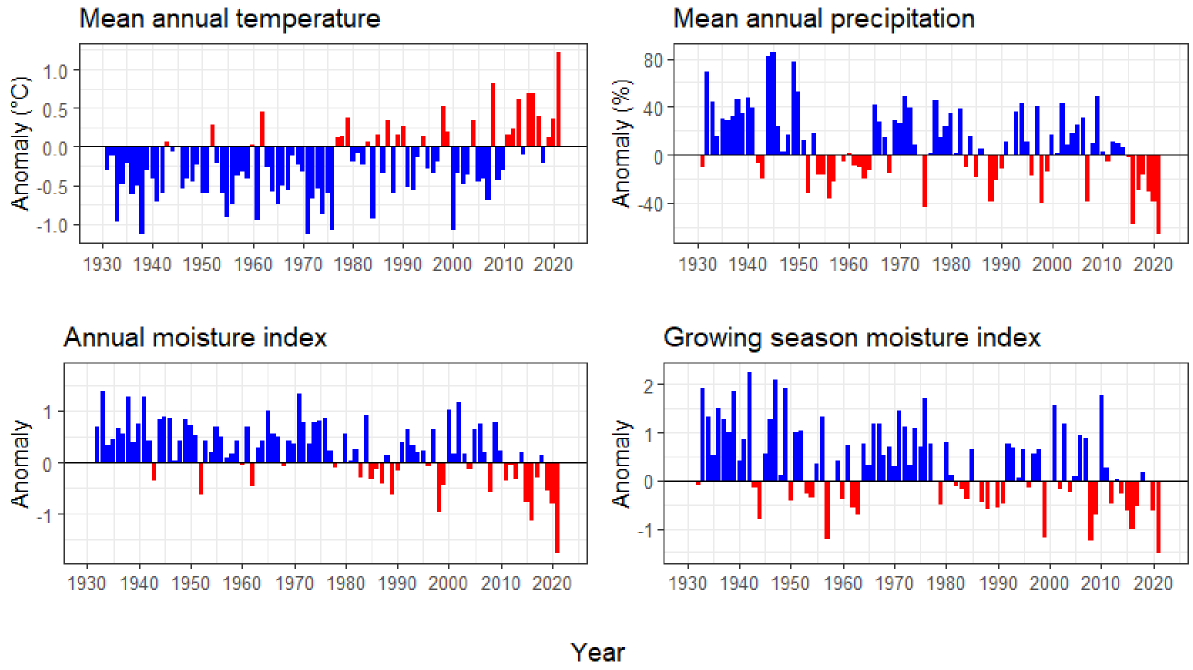

Figure S1. Regional climate series shown as anomalies relative to the period 1992-2021 computed for the period 1931-2021. Growing season values (computed for the period 1932-2021) include previous year October, November and December and current year January, February and March.
