# Supplementary material for: Losing half the crown hardly affects the stem growth of a xeric southern beech population
Source: Sci Rep. 2025 Feb 17;15:5721. doi: 10.1038/s41598-025-90061-9 (PMC11832943; doi:10.1038/s41598-025-90061-9)
Supplement: Supplementary file 2 — Supplementary Material 2 [file 41598_2025_90061_MOESM2_ESM.pdf]

Appendix II

Detrending and standardization of tree ring series

Our focus on maximizing high- and low- frequency climate signals for evaluating the climate growth correlation prompted us to explore the importance of different standardization and detrending techniques, based on the outcome of the trend analyses (divergent dominant patters among the same stand). Five methods available in the R package dplR were employed for the analysis of climate-growth correlations with different standardization and detrending methods of both, the RWL and the BAI series: (1) Spline30: a smoothing spline with a 50% frequency cut-off at 30 years (Cook & Peters, 1981); (2) Spline150: a smoothing spline with a 50% frequency cut-off at 150 years (equivalent to two third of the mean series length; Klesse, 2021); (3) Ar: residuals of an autoregressive model (Cook and Kairiukstis, 1990); (4) NegExp: modified negative exponential curve (Fritts, 1976); and (5) Mean: horizontal line equal to the series mean.

For each of the 6 chronologies assessed (whole-site chronology plus the subgroups PC1 and PC2, using either RWL or BAI as input data), the positive or negative influence of climate variables of a given month remained fairly consistent across the different methods used for standardization and detrending. However, the size of the correlation coefficients and the number of significantly correlated months to annual growth rate were noticeable influenced by the method used (Table S3).

Correlation coefficients between climatic variables and raw (unmodified) RWL and BAI data, and growth rates standardized and detrended with static and more conservative methods (Spline150, Mean, NegExp), showed higher Pearson r values than climate-growth relationships

that base on dynamic and more forceful detrending methods (Spline30 and Ar) (Fig. S2). In accordance, the number of months with significant climate correlation to growth was higher, when using either raw RWL data or values standardized with static methods (Mean and NegExp) (Fig. S2). However, high-frequency signals such as the positive influence of precipitation during current growing season December, were best captured when using the Ar method, regardless of whether applied to RWL or BAI data (Table S3).

Principally, all sampled trees (Site chronology), the ones following a positive BAI trend (PC1 group) and the ones with a negative BAI trend (PC2 group) revealed similar patterns of climatic drivers of growth. However, the extent of climate sensitivity as expressed by the size of the correlation coefficient and the number of months with significant correlations, differed between the three groups, irrespective of the detrending method used for the analysis. However, the group differences partly disappeared under the more forceful methods (Spl30 and Ar; Fig. S2).

Table S3: Pearson correlation coefficients between raw and detrended tree ring series (ring width length, RWL and basal area increment, BAI) and standardized monthly climate variables (Temperature, Precipitation and Moisture Index, from October of the previous growing season to April of the current growing season). Cells colored in red indicate negative, in green positive correlation coefficients. Numbers in bold indicate significant correlations at $p<0.05$.

Temperature - RWL

	Month	Site						PC1						PC2					
		Raw	Spl30	Spl150	Ar	Mean	NegExp	Raw	Spl30	Spl150	Ar	Mean	NegExp	Raw	Spl30	Spl150	Ar	Mean	NegExp
Previous growing season	oct	-0.199	-0.180	-0.178	-0.158	-0.181	-0.180	-0.141	-0.146	-0.144	-0.121	-0.150	-0.150	-0.188	-0.194	-0.181	**-0.214**	-0.185	-0.185
	nov	**-0.293**	**-0.232**	**-0.281**	**-0.248**	**-0.280**	**-0.276**	**-0.238**	-0.189	**-0.238**	-0.221	**-0.233**	**-0.233**	**-0.300**	**-0.289**	**-0.317**	**-0.259**	**-0.290**	**-0.290**
	dec	**-0.230**	-0.160	**-0.213**	-0.172	**-0.242**	**-0.233**	**-0.222**	-0.142	**-0.192**	-0.114	**-0.220**	**-0.220**	-0.159	**-0.167**	-0.164	-0.131	-0.150	-0.150
	Jan	-0.165	-0.061	-0.141	-0.108	**-0.181**	**-0.176**	**-0.198**	-0.051	-0.094	-0.085	-0.163	-0.163	-0.112	-0.109	-0.100	-0.118	-0.073	-0.073
	Feb	**-0.282**	-0.190	**-0.251**	-0.066	**-0.294**	**-0.290**	**-0.294**	-0.188	**-0.216**	-0.051	**-0.265**	**-0.265**	-0.113	-0.155	-0.132	0.029	-0.089	-0.089
	Mar	-0.080	0.062	-0.075	0.050	-0.103	-0.103	-0.108	0.093	-0.018	0.079	-0.075	-0.075	-0.065	0.017	-0.063	0.029	-0.043	-0.043
	Apr	0.139	0.150	0.146	0.085	0.138	0.142	0.119	0.134	0.109	0.069	0.116	0.116	0.106	0.137	0.094	0.022	0.093	0.093
	May	0.138	0.137	0.154	-0.003	0.142	0.145	0.136	0.155	0.140	0.015	0.125	0.125	0.123	0.136	0.143	0.004	0.129	0.129
	Jun	-0.080	-0.101	-0.083	-0.108	-0.087	-0.096	-0.077	-0.079	-0.045	-0.092	-0.055	-0.055	-0.008	-0.002	0.007	-0.007	0.004	0.004
	Jul	0.100	0.009	0.082	0.047	0.098	0.087	0.123	0.028	0.110	0.063	0.132	0.132	0.015	0.026	0.054	0.045	0.034	0.034
	Aug	0.038	0.059	0.048	-0.041	0.037	0.025	0.029	0.084	0.091	-0.016	0.062	0.062	0.101	0.075	0.114	0.019	0.106	0.106
	Sep	-0.085	-0.115	-0.087	-0.128	-0.112	-0.116	-0.128	-0.110	-0.072	-0.088	-0.099	-0.099	0.036	-0.082	0.027	-0.121	0.065	0.065
Growing season	Oct	-0.087	-0.050	-0.067	-0.016	-0.079	-0.088	-0.101	-0.042	-0.044	0.010	-0.065	-0.065	-0.039	0.009	-0.006	0.017	-0.009	-0.009
	Nov	-0.089	0.037	-0.061	0.105	-0.084	-0.083	-0.073	0.031	-0.042	0.109	-0.056	-0.056	-0.115	-0.036	-0.097	0.056	-0.094	-0.094
	Dec	**-0.182**	-0.065	-0.146	**-0.217**	**-0.185**	-0.176	**-0.231**	-0.082	-0.175	-0.208	**-0.216**	**-0.216**	-0.105	-0.064	-0.118	**-0.193**	-0.092	-0.092
	JAN	-0.131	-0.020	-0.106	-0.080	-0.139	-0.135	**-0.171**	-0.033	-0.082	-0.036	-0.145	-0.145	-0.045	-0.030	-0.031	-0.123	-0.005	-0.005
	FEB	**-0.303**	**-0.233**	**-0.291**	-0.206	**-0.333**	**-0.334**	**-0.371**	**-0.254**	**-0.282**	**-0.225**	**-0.341**	**-0.341**	-0.154	-0.142	-0.172	-0.123	-0.130	-0.130
	MAR	-0.141	0.001	-0.120	-0.013	-0.152	-0.144	-0.186	-0.005	-0.116	0.008	-0.175	-0.175	-0.066	-0.016	-0.102	-0.021	-0.070	-0.070
	APR	0.138	0.138	0.120	0.061	0.113	0.114	0.102	0.135	0.109	0.068	0.108	0.108	0.122	0.119	0.102	0.048	0.112	0.112

Precipitation - RWL

	Month	Site						PC1						PC2					
		Raw	Spl30	Spl150	Ar	Mean	NegExp	Raw	Spl30	Spl150	Ar	Mean	NegExp	Raw	Spl30	Spl150	Ar	Mean	NegExp
Previous growing season	oct	**0.166**	**0.182**	**0.172**	**0.210**	0.158	0.160	0.138	**0.176**	**0.194**	**0.210**	0.153	0.153	**0.247**	**0.231**	**0.272**	**0.300**	**0.274**	**0.274**
	nov	**0.173**	0.158	**0.173**	0.165	0.174	0.162	0.117	0.122	0.138	0.129	0.113	0.113	**0.229**	**0.248**	**0.243**	**0.213**	**0.239**	**0.239**
	dec	0.105	0.078	0.097	0.030	0.113	0.112	0.070	0.070	0.055	-0.003	0.057	0.057	0.075	0.072	0.059	0.015	0.062	0.062
	Jan	0.102	0.061	0.106	0.048	0.113	0.117	0.098	0.046	0.077	0.024	0.094	0.094	0.111	0.089	0.106	0.062	0.104	0.104
	Feb	0.019	-0.020	0.010	-0.075	0.034	0.030	0.022	-0.011	0.016	-0.084	0.027	0.027	-0.001	-0.032	0.011	-0.139	0.001	0.001
	Mar	0.089	-0.023	0.070	-0.051	0.092	0.086	0.142	-0.017	0.093	-0.044	0.126	0.126	0.059	-0.056	0.065	-0.042	0.048	0.048
	Apr	0.158	0.116	0.157	0.189	0.152	0.154	0.209	0.192	0.229	**0.273**	**0.229**	**0.229**	0.095	0.092	0.121	0.172	0.111	0.111
	May	0.156	0.149	0.133	0.047	0.155	0.160	0.130	0.131	0.092	0.022	0.114	0.114	0.125	0.121	0.112	-0.001	0.103	0.103
	Jun	**0.232**	**0.150**	**0.219**	0.124	**0.215**	**0.215**	**0.186**	**0.174**	**0.225**	0.110	**0.205**	**0.205**	**0.248**	0.146	**0.242**	0.094	**0.233**	**0.233**
	Jul	**0.294**	0.183	**0.284**	0.199	**0.292**	**0.295**	**0.319**	0.188	**0.278**	0.209	**0.313**	**0.313**	0.179	0.147	0.205	0.125	0.174	0.174
	Aug	0.038	-0.006	0.026	0.021	0.040	0.038	0.050	0.016	0.045	0.045	0.045	0.045	-0.056	-0.033	-0.042	-0.032	-0.045	-0.045
	Sep	0.070	0.095	0.057	-0.010	0.072	0.071	0.074	0.104	0.050	0.037	0.059	0.059	0.061	0.134	0.085	0.003	0.067	0.067
Growing season	Oct	-0.022	-0.068	-0.036	-0.069	-0.036	-0.035	-0.028	-0.045	-0.027	-0.052	-0.025	-0.025	-0.002	-0.093	-0.036	-0.069	-0.020	-0.020
	Nov	0.046	-0.019	0.025	-0.082	0.035	0.032	0.013	-0.012	0.017	-0.102	0.009	0.009	0.082	0.072	0.068	0.016	0.088	0.088
	Dec	**0.178**	0.132	0.150	**0.269**	**0.176**	0.177	0.171	0.117	0.152	**0.241**	0.168	0.168	0.118	0.120	0.136	**0.237**	0.130	0.130
	JAN	0.115	0.059	0.108	0.089	0.112	0.108	0.102	0.052	0.077	0.060	0.103	0.103	0.101	0.075	0.086	0.138	0.072	0.072
	FEB	0.102	0.066	0.096	0.049	0.103	0.099	0.096	0.053	0.081	0.040	0.094	0.094	0.043	0.027	0.074	0.025	0.064	0.064
	MAR	0.164	0.068	0.140	0.044	0.165	0.161	0.207	0.086	0.176	0.056	0.215	0.215	0.113	0.042	0.142	0.005	0.126	0.126
	APR	-0.002	0.015	-0.008	0.034	0.002	0.004	0.041	0.051	0.062	0.117	0.054	0.054	-0.054	-0.011	-0.014	0.021	-0.017	-0.017

Moisture Index - RWL

	Month	Site						PC1						PC2					
		Raw	Spl30	Spl150	Ar	Mean	NegExp	Raw	Spl30	Spl150	Ar	Mean	NegExp	Raw	Spl30	Spl150	Ar	Mean	NegExp
Previous growing season	oct	**0.212**	**0.210**	**0.203**	**0.203**	**0.197**	**0.202**	**0.167**	**0.181**	**0.191**	0.176	**0.176**	**0.176**	**0.240**	**0.239**	**0.250**	**0.286**	**0.250**	**0.250**
	nov	**0.269**	**0.225**	**0.263**	**0.246**	**0.264**	**0.249**	**0.201**	0.186	**0.211**	0.205	**0.198**	**0.198**	**0.303**	**0.312**	**0.324**	**0.273**	**0.307**	**0.307**
	dec	0.181	0.131	0.164	0.107	0.196	**0.186**	0.159	0.115	0.132	0.064	0.147	0.147	0.121	0.130	0.122	0.079	0.109	0.109
	Jan	0.153	0.073	0.142	0.094	0.180	0.175	0.176	0.055	0.101	0.067	0.151	0.151	0.128	0.111	0.120	0.111	0.101	0.101
	Feb	0.182	0.102	0.161	-0.004	0.203	0.194	0.192	0.110	0.140	-0.020	0.179	0.179	0.068	0.075	0.084	-0.101	0.053	0.053
	Mar	0.114	-0.043	0.098	-0.065	0.134	0.127	0.169	-0.059	0.073	-0.075	0.139	0.139	0.086	-0.036	0.097	-0.040	0.065	0.065
	Apr	0.023	-0.019	0.020	0.078	0.022	0.021	0.067	0.052	0.092	0.146	0.087	0.087	0.008	-0.019	0.036	0.110	0.020	0.020
	May	-0.011	-0.016	-0.039	0.024	-0.011	-0.010	-0.017	-0.040	-0.059	-0.004	-0.023	-0.023	-0.021	-0.038	-0.050	-0.020	-0.050	-0.050
	Jun	**0.246**	**0.192**	**0.235**	0.174	**0.234**	**0.243**	**0.212**	**0.193**	**0.207**	0.148	**0.198**	**0.198**	**0.190**	0.107	0.182	0.065	0.180	0.180
	Jul	0.155	0.137	0.149	0.131	0.150	0.161	0.153	0.120	0.124	0.132	0.140	0.140	0.142	0.104	0.127	0.079	0.127	0.127
	Aug	-0.006	-0.048	-0.021	0.043	0.002	0.002	0.015	-0.054	-0.036	0.040	-0.012	-0.012	-0.117	-0.087	-0.121	-0.045	-0.124	-0.124
	Sep	0.104	0.143	0.107	0.086	0.129	0.128	0.139	0.147	0.085	0.092	0.113	0.113	0.021	0.149	0.039	0.089	0.009	0.009
Growing season	Oct	0.052	0.007	0.027	-0.007	0.046	0.051	0.066	0.015	0.025	-0.019	0.043	0.043	0.034	-0.045	-0.003	-0.034	-0.003	-0.003
	Nov	0.072	-0.034	0.045	-0.111	0.065	0.060	0.044	-0.027	0.030	-0.125	0.029	0.029	0.107	0.058	0.086	-0.027	0.100	0.100
	Dec	**0.193**	0.106	0.158	**0.259**	**0.194**	**0.192**	**0.220**	0.108	0.174	**0.244**	**0.207**	**0.207**	0.117	0.094	0.137	**0.233**	0.113	0.113
	JAN	0.138	0.037	0.117	0.092	0.141	0.141	0.160	0.042	0.087	0.055	0.143	0.143	0.080	0.054	0.063	0.153	0.035	0.035
	FEB	**0.247**	0.181	**0.237**	0.155	**0.270**	**0.263**	**0.290**	0.187	0.226	0.163	**0.267**	**0.267**	0.119	0.099	0.148	0.086	0.116	0.116
	MAR	0.205	0.055	0.180	0.046	**0.212**	0.208	**0.257**	0.067	0.194	0.039	**0.254**	**0.254**	0.121	0.049	0.166	0.024	0.127	0.127
	APR	-0.096	-0.084	-0.087	-0.022	-0.075	-0.071	-0.047	-0.057	-0.038	0.021	-0.044	-0.044	-0.113	-0.087	-0.081	-0.023	-0.086	-0.086

	Month	Temperature - BAI																	
		Site						PC1						PC2					
		Raw	Spl30	Spl150	Ar	Mean	NegExp	Raw	Spl30	Spl150	Ar	Mean	NegExp	Raw	Spl30	Spl150	Ar	Mean	NegExp
Previous growing season	oct	-0.167	-0.187	-0.184	-0.150	-0.184	-0.188	-0.150	**-0.216**	**-0.209**	-0.177	-0.181	-0.175	-0.185	-0.098	-0.072	-0.054	-0.115	-0.120
	nov	**-0.270**	-0.226	**-0.272**	-0.230	**-0.263**	**-0.268**	**-0.233**	**-0.254**	**-0.294**	-0.238	**-0.254**	**-0.251**	**-0.290**	-0.133	-0.109	-0.180	-0.183	-0.187
	dec	-0.150	-0.157	-0.206	-0.148	-0.192	**-0.192**	**-0.220**	-0.189	**-0.229**	-0.164	-0.157	-0.154	-0.150	-0.083	-0.094	-0.103	-0.202	-0.197
	Jan	-0.085	-0.058	-0.131	-0.074	-0.112	-0.108	-0.163	-0.087	-0.146	-0.090	-0.051	-0.051	-0.073	0.013	-0.036	-0.037	**-0.207**	**-0.203**
	Feb	-0.167	-0.190	**-0.229**	-0.024	-0.212	-0.211	**-0.265**	-0.223	**-0.256**	-0.038	-0.136	-0.129	-0.089	-0.102	-0.122	-0.021	**-0.344**	**-0.352**
	Mar	0.004	0.058	-0.049	0.100	-0.016	-0.018	-0.075	0.047	-0.056	0.119	0.043	0.041	-0.043	0.057	-0.009	0.007	-0.164	-0.149
	Apr	0.134	0.157	0.142	0.094	0.150	0.150	0.116	0.129	0.114	0.059	0.129	0.124	0.093	0.233	**0.234**	0.144	0.154	0.160
	May	0.146	0.140	0.156	-0.003	0.152	0.157	0.125	0.128	0.139	-0.027	0.141	0.137	0.129	0.155	**0.179**	0.025	0.115	0.128
	Jun	-0.088	-0.104	-0.084	-0.087	-0.096	-0.093	-0.055	-0.100	-0.076	-0.063	-0.083	-0.096	0.004	-0.082	-0.060	-0.104	-0.064	-0.071
	Jul	0.061	0.005	0.079	0.018	0.049	0.069	0.132	0.016	0.077	-0.001	0.029	0.028	0.034	0.012	0.081	0.068	0.126	0.116
	Aug	0.058	0.055	0.049	-0.041	0.047	0.054	0.062	0.039	0.041	-0.029	0.055	0.050	0.106	0.098	0.094	-0.041	0.010	0.014
	Sep	-0.048	-0.120	-0.086	-0.127	-0.065	-0.070	-0.099	-0.095	-0.053	-0.112	-0.004	0.002	0.065	-0.180	**-0.203**	**-0.199**	**-0.241**	**-0.240**
Growing season	Oct	-0.045	-0.052	-0.072	-0.013	-0.064	-0.063	-0.065	-0.029	-0.064	-0.003	-0.045	-0.038	-0.009	-0.084	-0.091	-0.013	-0.084	-0.080
	Nov	-0.062	0.024	-0.051	0.130	-0.041	-0.048	-0.056	0.001	-0.084	0.127	-0.032	-0.024	-0.094	0.062	0.022	0.097	-0.053	-0.054
	Dec	-0.104	-0.059	-0.138	-0.175	-0.120	-0.119	**-0.216**	-0.072	-0.152	-0.203	-0.079	-0.078	-0.092	-0.018	-0.066	-0.139	-0.186	-0.171
	JAN	-0.019	-0.008	-0.094	-0.048	-0.044	-0.045	-0.145	-0.015	-0.099	-0.053	0.023	0.024	-0.005	0.023	-0.058	-0.069	**-0.213**	**-0.215**
	FEB	-0.183	**-0.237**	**-0.276**	-0.141	-0.213	-0.212	**-0.341**	**-0.222**	**-0.258**	-0.120	-0.112	-0.114	-0.130	**-0.243**	**-0.269**	**-0.262**	**-0.445**	**-0.443**
	MAR	-0.037	-0.004	-0.112	0.038	-0.062	-0.061	-0.175	-0.025	-0.120	0.040	-0.014	-0.010	-0.070	0.014	-0.063	-0.059	-0.204	-0.186
	APR	0.142	0.130	0.128	0.065	0.148	0.141	0.108	0.145	0.125	0.051	0.139	0.149	0.112	0.063	0.106	0.045	0.042	0.045

	Month	Precipitation -BAI																	
		Site						PC1						PC2					
		Raw	Spl30	Spl150	Ar	Mean	NegExp	Raw	Spl30	Spl150	Ar	Mean	NegExp	Raw	Spl30	Spl150	Ar	Mean	NegExp
Previous growing season	oct	**0.213**	**0.183**	**0.168**	**0.228**	**0.195**	**0.197**	0.153	**0.196**	**0.196**	**0.247**	**0.222**	**0.223**	**0.274**	0.142	0.089	0.091	0.022	0.030
	nov	**0.219**	0.160	**0.179**	0.175	**0.195**	**0.196**	0.113	**0.187**	**0.193**	0.187	**0.230**	**0.232**	**0.239**	0.078	0.062	0.055	0.021	0.035
	dec	0.069	0.077	0.099	0.013	0.090	0.090	0.057	0.096	0.108	0.014	0.088	0.090	0.062	0.012	0.010	0.025	0.069	0.065
	Jan	0.100	0.064	0.098	0.037	0.097	0.099	0.094	0.073	0.109	0.033	0.096	0.099	0.104	0.014	0.025	0.028	0.070	0.063
	Feb	-0.009	-0.018	0.012	-0.092	0.000	0.002	0.027	-0.008	0.032	-0.082	0.010	0.005	0.001	-0.023	-0.024	-0.073	0.026	0.031
	Mar	-0.002	-0.031	0.049	-0.087	0.027	0.017	0.126	-0.030	0.058	-0.100	-0.030	-0.020	0.048	-0.009	0.005	-0.016	0.126	0.124
	Apr	0.116	0.116	0.146	0.148	0.122	0.116	**0.229**	0.104	0.145	0.134	0.093	0.086	0.111	0.111	0.105	0.212	0.157	0.157
	May	0.098	0.156	0.146	0.017	0.108	0.105	0.114	0.148	0.125	-0.015	0.053	0.059	0.103	0.148	0.133	0.122	**0.222**	**0.220**
	Jun	**0.216**	**0.156**	**0.220**	0.107	**0.205**	**0.199**	**0.205**	0.170	**0.249**	0.140	**0.226**	**0.225**	**0.233**	0.082	0.052	0.014	0.065	0.063
	Jul	0.207	0.180	**0.269**	0.153	**0.242**	0.235	**0.313**	0.182	**0.282**	0.122	0.176	0.183	0.174	0.154	0.204	**0.219**	**0.309**	**0.312**
	Aug	0.029	-0.002	0.023	0.016	0.010	0.018	0.045	-0.019	0.018	-0.017	-0.004	-0.002	-0.045	0.038	0.081	0.090	0.078	0.075
	Sep	0.027	0.095	0.054	-0.035	0.028	0.032	0.059	0.078	0.028	-0.062	-0.016	-0.021	0.067	0.155	0.081	0.107	0.146	0.141
Growing season	Oct	-0.021	-0.061	-0.038	-0.075	-0.034	-0.036	-0.025	-0.081	-0.028	-0.070	-0.012	-0.012	-0.020	-0.018	-0.050	-0.034	-0.062	-0.056
	Nov	0.052	-0.012	0.035	-0.084	0.033	0.038	0.009	0.018	0.059	-0.066	0.058	0.056	0.088	-0.069	-0.054	-0.076	-0.042	-0.031
	Dec	0.157	0.121	0.148	**0.245**	0.156	0.152	0.168	0.151	0.170	**0.261**	0.155	0.147	0.130	0.057	0.049	0.181	0.116	0.101
	JAN	0.089	0.049	0.101	0.076	0.090	0.088	0.103	0.084	0.115	0.099	0.097	0.099	0.072	-0.012	0.036	0.045	0.049	0.051
	FEB	0.067	0.060	0.084	0.018	0.069	0.075	0.094	0.072	0.099	0.028	0.047	0.050	0.064	0.064	0.028	0.056	0.116	0.108
	MAR	0.087	0.075	0.124	0.022	0.111	0.100	0.215	0.072	0.147	0.029	0.059	0.059	0.126	0.085	0.100	0.047	0.194	0.187
	APR	-0.003	0.013	-0.011	0.048	-0.012	-0.010	0.054	0.016	0.003	0.057	-0.005	-0.012	-0.017	0.018	-0.034	0.008	0.000	0.003

	Month	Moisture Index - BAI																	
		Site						PC1						PC2					
		Raw	Spl30	Spl150	Ar	Mean	NegExp	Raw	Spl30	Spl150	Ar	Mean	NegExp	Raw	Spl30	Spl150	Ar	Mean	NegExp
Previous growing season	oct	**0.211**	**0.208**	**0.199**	**0.205**	**0.217**	**0.215**	**0.176**	**0.237**	**0.233**	**0.228**	**0.224**	**0.221**	**0.250**	0.139	0.096	0.083	0.092	0.100
	nov	**0.280**	**0.226**	**0.265**	0.237	**0.269**	**0.271**	**0.198**	**0.264**	**0.285**	**0.252**	**0.285**	**0.273**	**0.307**	0.128	0.099	0.138	0.124	0.130
	dec	0.115	0.124	0.166	0.088	0.153	0.152	0.147	0.155	0.180	0.093	0.129	0.129	0.109	0.053	0.060	0.074	0.152	0.148
	Jan	0.104	0.072	0.135	0.067	0.117	0.118	0.151	0.096	0.150	0.073	0.080	0.081	0.101	-0.003	0.033	0.044	0.165	0.160
	Feb	0.101	0.109	0.145	-0.039	0.131	0.131	0.179	0.133	0.174	-0.023	0.083	0.081	0.053	0.050	0.057	-0.023	**0.236**	**0.239**
	Mar	0.007	-0.049	0.071	-0.113	0.034	0.033	0.139	-0.036	0.080	-0.137	-0.036	-0.031	0.065	-0.033	0.016	-0.012	0.194	0.180
	Apr	-0.008	-0.014	0.007	0.045	-0.007	-0.011	0.087	-0.001	0.037	0.059	-0.018	-0.015	0.020	-0.073	-0.077	0.055	0.005	0.005
	May	-0.053	-0.018	-0.032	0.002	-0.053	-0.062	-0.023	-0.010	-0.029	0.004	-0.089	-0.074	-0.050	-0.029	-0.062	0.065	0.061	0.050
	Jun	**0.230**	**0.198**	**0.231**	0.142	**0.230**	**0.226**	**0.198**	**0.207**	**0.250**	0.156	**0.239**	**0.238**	0.180	0.132	0.094	0.093	0.117	0.108
	Jul	0.118	0.128	0.148	0.118	0.150	0.134	0.140	0.125	0.156	0.104	0.110	0.113	0.127	0.114	0.096	0.125	0.149	0.148
	Aug	-0.034	-0.054	-0.022	0.034	-0.030	-0.038	-0.012	-0.053	-0.028	0.006	-0.054	-0.051	-0.124	-0.044	-0.009	0.085	0.053	0.047
	Sep	0.058	0.147	0.097	0.067	0.072	0.076	0.113	0.116	0.060	0.039	-0.005	-0.008	0.009	**0.224**	0.189	**0.212**	**0.260**	**0.263**
Growing season	Oct	0.022	0.015	0.036	-0.015	0.033	0.029	0.043	-0.013	0.034	-0.020	0.027	0.022	-0.003	0.051	0.043	0.008	0.036	0.030
	Nov	0.063	-0.023	0.046	-0.124	0.042	0.043	0.029	0.005	0.077	-0.116	0.055	0.047	0.100	-0.074	-0.048	-0.107	0.000	0.006
	Dec	0.138	0.094	0.154	**0.229**	0.143	0.144	**0.207**	0.117	0.170	0.251	0.122	0.115	0.113	0.039	0.062	0.174	0.171	0.154
	JAN	0.049	0.025	0.105	0.069	0.067	0.070	0.143	0.053	0.119	0.087	0.031	0.030	0.035	-0.034	0.045	0.059	0.161	0.159
	FEB	0.151	0.179	**0.218**	0.093	0.171	0.175	**0.267**	0.177	0.218	0.091	0.100	0.100	0.116	0.185	0.178	0.194	**0.344**	**0.339**
	MAR	0.089	0.060	0.161	-0.002	0.121	0.113	**0.254**	0.072	0.181	0.002	0.052	0.052	0.127	0.057	0.112	0.072	**0.261**	**0.250**
	APR	-0.100	-0.076	-0.095	-0.020	-0.107	-0.101	-0.044	-0.088	-0.084	-0.002	-0.095	-0.100	-0.086	-0.028	-0.093	-0.029	-0.027	-0.035

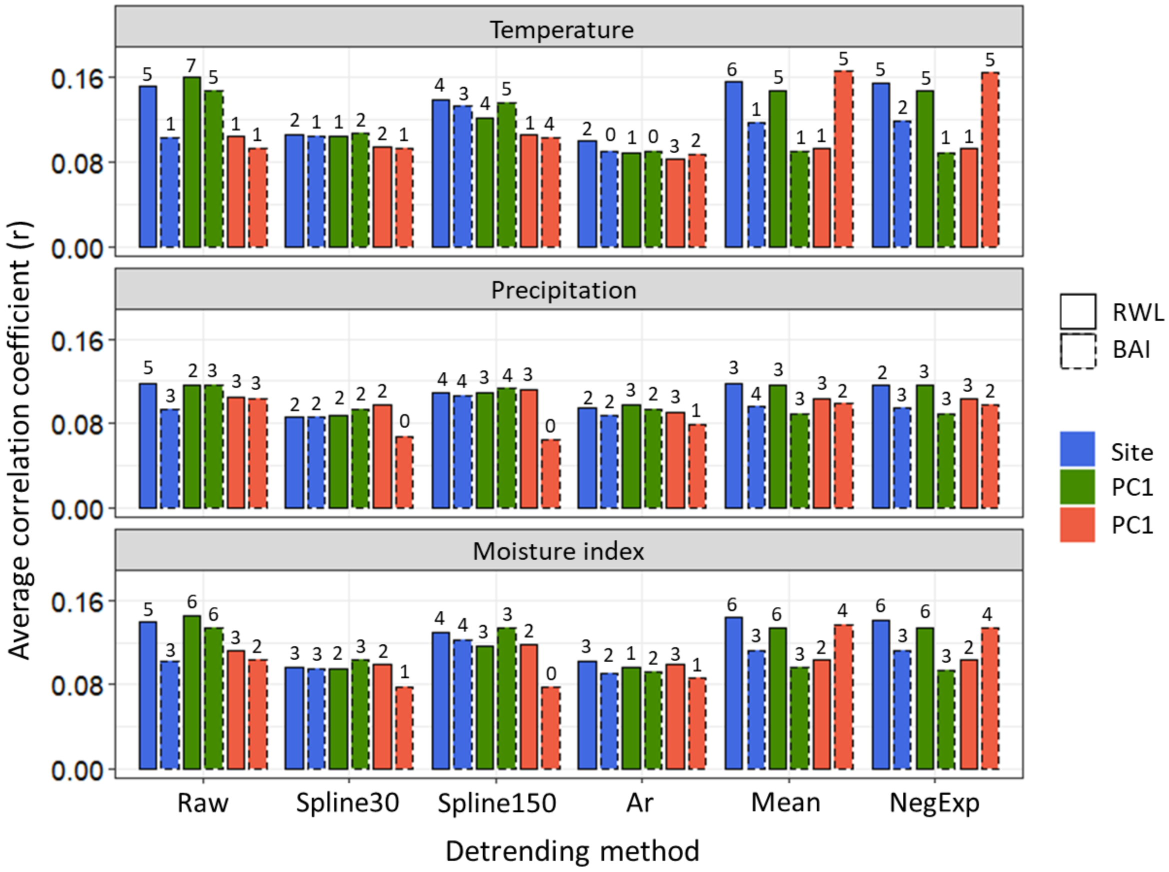

Figure S2. Average Pearson correlation coefficients for the relationships between ring width length values (RWL; solid bars) or basal area increment (BAI, dashed bars) and mean monthly temperature (upper panel), monthly precipitation (middle panel) and monthly moisture index (lower panel) in the period 1933-2021 for ring width data subjected to six different detrending methods in the full tree sample of 23 trees: Site chronology (blue bars), or the sub-samples of trees associated to PC1 (green bars) or PC2 (red bars). The correlation analyses were conducted for the 19 months in the years prior and during growth; the bars give absolute r values averaged over the 19 months. Numbers on top of the bars give the number of months with significant ($p<0.05$) correlation. The six detrending methods are: Raw - no standardization and detrending; Spline30 - smoothing spline with a 50% frequency cut-off at 30 years; Spline150- smoothing spline with a 50% frequency cut-off at 150 years; Ar - residuals of an autoregressive model; Mean - horizontal line equal to the series mean; NegExp - modified negative exponential curve
